# Isolation and Purification of Chitosan Oligosaccharides (Mw ≤ 1000) and Their Protective Effect on Acute Liver Injury Caused by CCl_4_

**DOI:** 10.3390/md22030128

**Published:** 2024-03-08

**Authors:** Kai Wang, Dawei Yu, Yan Bai, Hua Cao, Jiao Guo, Zhengquan Su

**Affiliations:** 1 Guangdong Provincial University Engineering Technology Research Center of Natural Products and Drugs, Guangdong Pharmaceutical University, Guangzhou 510006, China; wk972023826@163.com (K.W.); ydw65038@163.com (D.Y.); 2 Guangdong Metabolic Disease Research Center of Integrated Chinese and Western Medicine, Guangdong Pharmaceutical University, Guangzhou 510006, China; 3 School of Public Health, Guangdong Pharmaceutical University, Guangzhou 510310, China; angell_bai@163.com; 4 School of Chemistry and Chemical Engineering, Guangdong Pharmaceutical University, Zhongshan 528458, China; caohua@gdpu.edu.cn

**Keywords:** acute liver injury, CCl_4_, chitosan oligosaccharide, oxidation resistance

## Abstract

Chitosan oligosaccharides are the degradation products of chitin obtained from the shell extracts of shrimps and crabs. Compared with chitosan, chitosan oligosaccharides have better solubility and a wider application range. In this study, high-molecular-weight chitosan oligosaccharides (COST, chitosan oligosaccharides, MW ≤ 1000) were isolated and purified by a GPC gel column, and the molecular weight range was further reduced to obtain high-purity and low-molecular-weight chitosan (COS46). Compared with COST, COS46 is better at inhibiting CCl_4_-induced cell death, improving cell morphology, reducing ALT content, and improving cell antioxidant capacity. The effects of COST and COS46 on CCl_4_-induced acute liver injury were further verified in mice. Both COS46 and COST improved the appearance of the liver induced by CCl_4_, decreased the levels of ALT and AST in serum, and decreased the oxidation/antioxidant index in the liver. From the liver pathological section, the effect of COS46 was better. In addition, some indicators of COS46 showed a dose-dependent effect. In conclusion, compared with COST, low-molecular-weight COS46 has better antioxidant capacity and a better therapeutic effect on CCl_4_-induced acute liver injury.

## 1. Introduction

At present, the shells of crabs and shrimps in marine arthropods are often ignored and underutilized, which are important sources of chitin. After a series of compound chemical treatments, chitosan and chitosan oligosaccharides are obtained, which have important application value in medical care and food [1]. Chitosan oligosaccharide itself has a variety of physiological activities, such as antibacterial properties [2,3], anti-tumor properties [4,5], antioxidant [3,6] and anti-inflammatory effects [7], etc. There are also studies using chitosan oligosaccharides as a drug carrier [8,9] or as a prebiotic to explore their influence on intestinal flora in different diseases [10,11]. It has also been revealed to have anti-diabetes [12], anti-obesity [13], anti-HIV-1 [14], and anti-Alzheimer’s disease [15] effects and promote calcium absorption [16].

The excellent antioxidant properties of chitosan oligosaccharides have been the focus of related research, and it has been proven that it can reduce oxidative stress injury by scavenging free radicals and reactive oxygen species and maintaining the activity of antioxidant enzymes [17,18]. Therefore, the antioxidant properties of chitosan oligosaccharides can be identified from two aspects: the ability to scavenge free radicals and the ability to improve the level of antioxidant substances in vivo. However, the exact mechanism has not been explained. According to existing studies and the chemical structure of chitosan oligosaccharides, it is speculated that its amino group can combine with unstable free radicals to form a stable structure, which shows antioxidant properties.

Recent studies have indicated that the biological activity of chitosan oligosaccharides is primarily influenced by DP (degree of polymerization) and Mw (molecular weight). Therefore, it is difficult to determine which parts of chitosan oligosaccharides play a major role in biological activity [19]. Although there is no exact evidence to directly show the relationship between chitosan oligosaccharides’ antioxidant properties and molecular weight, it is speculated from the existing relevant studies that a low molecular weight and a high degree of deacetylation can equip chitosan oligosaccharides with a better antioxidant effect [20], and the relationship between the two needs to be further explored.

Currently, chitosan oligosaccharides available in the market are typically synthesized from monosaccharides or extracted from hydrolyzed products. Researchers selectively synthesize the necessary oligosaccharides through specific chemical or enzymatic reactions. However, the synthesis process involves numerous steps and is costly. Therefore, it is more convenient to extract chitosan oligosaccharides [21,22,23]. Combined with the relationship between the properties of chitosan oligosaccharides and molecular weight, it is of great significance to narrow the molecular weight range through specific technical means, and even obtain chitosan oligosaccharides with a specific degree of polymerization. It is important not only to clarify the pharmacodynamic mechanism of chitosan oligosaccharides with different polymerization degrees but also to develop and utilize chitosan oligosaccharides more effectively. Due to the small difference in charge density and molecular weight between chitosan oligosaccharide monomers, the separation and purification of chitosan oligosaccharides are very challenging, especially when the degree of polymerization is greater than 4. This makes it a hotspot in the field of sugar science.

In the existing research, many methods for recovering and purifying chitosan oligosaccharides, such as gel filtration or size exclusion chromatography, have been found to purify and obtain specific chitosan oligosaccharides, and the molecular weight difference between different polymerization degrees of chitosan oligosaccharides is the basis for separation. It has been reported that chitosan oligosaccharides with a polymerization degree of 2–6 were separated from the hydrolysate of chitosan by gel filtration chromatography and ion exchange chromatography [24]. In an experiment of enzymatic hydrolysis of chitosan, the separation ability of size-exclusion chromatography (SEC) was used to help researchers fully understand the composition of the enzymatic hydrolysate [25]. Ultrafiltration [26] and nanofiltration [27] are also applied to the purification of chitosan oligosaccharides. Ion exchange chromatography [28] and metal affinity chromatography [29] are also widely used to detect and separate chitosan oligosaccharides.

High-performance liquid chromatography (HPLC) is often used for chitosan oligosaccharide analysis and purification [30]. HPLC is often combined with an ultraviolet detector, and the acetyl amino group of chitosan oligosaccharides can form a conjugated structure with the sugar ring, and the absorbance of chitosan oligosaccharides can be detected at a wavelength of approximately 210 nm. A differential refraction detector is preferred for the HPLC analysis of N-acetylated and N-deacetylated chitosan oligosaccharides, but its sensitivity is low and it is not suitable for gradient washing [31]. It is difficult to separate chitosan oligosaccharides by conventional HPLC, mainly because of its strong hydrophilicity and poor retention on HPLC columns [32]. Hydrophilic-interaction chromatography (HILIC) is a technique that can effectively separate polar and hydrophilic compounds. Some studies have successfully separated and purified chitosan oligosaccharides with a polymerization degree of 2–6 from a chitosan oligosaccharides mixture by using HILIC technology and used it to judge the influence of each component on RSC96 cells [32].

N- acetyl chitotriose and N, N’- diacetyl chitotriose can be separated from acetylated chitotriose by a cation exchange gel column [21]. For partially acetylated chitosan oligosaccharides, HPLC-ESI-MS can be used for separation and detection [33].

Capillary Electrophoresis (CE) has the advantage of high separation ability. Compared with HPLC, it has the characteristics of less sample loading, but it is precisely because of the small sample loading that a large number of chitosan oligosaccharides cannot be obtained by CE technology. When used to separate and detect chitosan oligosaccharides, electrophoretic mobility depends on the number of unit structures in an acidic aqueous solution, which is similar to other oligomeric electrolytes [28,34]. In many studies, chitosan oligosaccharides have been successfully separated using this technique [34,35].

CCl_4_ is a typical substance with hepatotoxicity, which will remove halogen atoms in liver microsomes through a redox reaction to generate the trichloromethyl radical and its derivative trichloromethyl peroxyradical active intermediate. These free radicals bind to phospholipid molecules on the endoplasmic reticulum (ER) through covalent bonds. This can inhibit protein synthesis, destroy the function and morphology of the cell membrane, and cause hepatotoxicity, fibrosis, lipid peroxidation, and ERS (Endoplasmic reticulum stress) reaction [36,37].

Acute liver injury induced by CCl_4_ in animals is very similar to acute chemical liver injury in humans, so it is widely used in the study of potential hepatoprotective drug activity [38]. CCl_4_ not only exerts strong toxicity on the liver but also causes damage to other parts of the body [39], including the kidneys [40], nervous system [41], and testes [42].

Currently, there is no research on the role of DP4-6 chitosan oligosaccharides in liver metabolic diseases. This paper proposes a hypothesis that the antioxidant properties of chitosan oligosaccharides are related to their molecular weight. It is speculated that low-molecular-weight chitosan oligosaccharides have a better protective effect on the liver. The paper also describes the process of obtaining DP4-6 chito-oligosaccharides by separating and purifying COST and named the product COS46. The hypothesis was confirmed through cell and animal experiments. The study investigated the difference in antioxidant properties and efficacy of COST in protecting against liver injury before and after separation. These findings are expected to aid in the further development and utilization of chitosan oligosaccharides.

## 2. Results

### 2.1. Isolation and Purification of COST

#### 2.1.1. COST Component and DP4-6 COS Content

**Figure 1:**
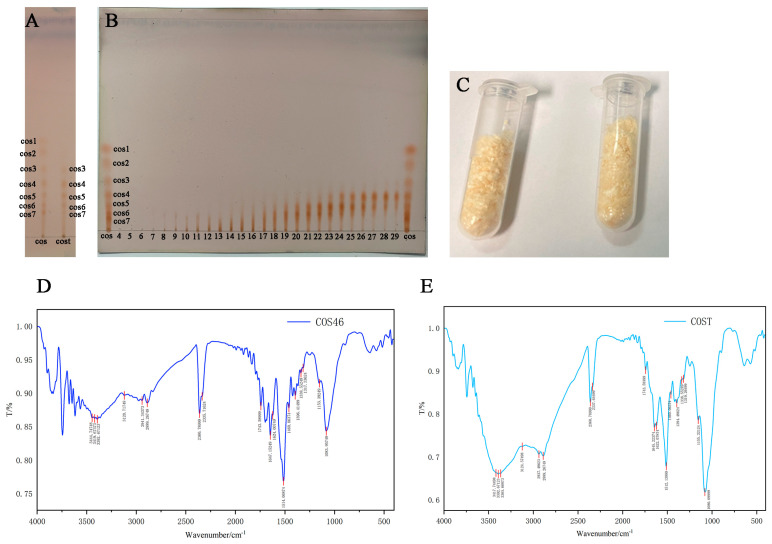
Thin layer diagram of COST and COS (Chitosan oligosaccharides mixed standard DP 1−7) (**A**); TLC thin-layer chromatography at different time periods. Serial number of collected eluate (4−29) (**B**); isolated COS46 freeze-dried powder (**C**); COS46 infrared spectrum (**D**); COST infrared spectrum (**E**).

The results of the TLC analysis of COST are shown in Figure 1A. The selected developer system has a good separation effect on COST, with obvious separation at each point and no obvious tailing phenomenon. According to TLC, compared with the mixed standard (1–7), COST contains chitosan oligosaccharides with a degree of polymerization of 3–7, but there are still a large number of substances piled up at the starting line of the thin layer, which cannot be separated. It is speculated that chitosan oligosaccharides with a higher degree of polymerization than chito-seven sugars exist in COST.

As shown in Table S1, the retention times of COS4, COS5, and COS6 are approximately 6 min, 8 min, and 12 min, respectively, and the standard curve is drawn according to the peak area of the concentration of each chitosan oligosaccharide standard series. The target principal component of COST is integral, and the peak area was 6166, 2267, and 3521, which was substituted into the standard curve for calculation. See the Supplementary Materials for the LC-MS spectrum and data on each standard product and COST (Figure S7, Table S2).

The component concentration of DP = 4(COS4) in COST was 0.99 mg/mL, and the content was 28.78%. The concentration of DP = 5(COS5) was 0.37 mg/mL and the content was 10.76%. The component concentration of DP = 6(COS6) was 0.61 mg/mL and the content was 17.73%. Therefore, in the COST of raw materials, the component content of DP4-6(COS46) is 57.27%.

#### 2.1.2. Gel Chromatographic Separation Yield

TLC chromatography of each tube is shown in Figure 1B. Chitosan oligosaccharides of the target polymerization degree appeared from 19#. The lyophilized product was light yellow, as shown in Figure 1C. As can be seen from Table 1, when the sample size at COST is approximately 0.3 g, approximately 0.1 g of COS46 can be harvested. The COS46 obtained by five times of separation is calculated, and the yield is 30.39 ± 1.57%.

#### 2.1.3. COS46 Purity and Infrared Spectrum

The target component of COS46 was also integrated, and the peak area was 4524, 2340, and 2783, which was substituted into the standard curve for calculation. See the Supplementary Materials for the LC-MS atlas and data on COS46 (Figure S6, Table S2).

In COS46, the component concentration of COS4 was 0.73 mg/mL and the content was 40.11%. The concentration of COS5 was 0.39 mg/mL and the content was 21.43%. The component concentration of COS6 was 0.49 mg/mL and the content was 26.92%. Therefore, the total proportion of the target component in COS46 was 88.46%.

The infrared spectra of COS46 and COST are shown in Figure 1D,E. Compared with the COST of raw materials, COS46 shows an obvious peak shape change at 3400 cm^−1^, where infrared absorption is mainly generated by hydrogen bonds. It is speculated that the reason is that the polymerization degree of COS46 decreases, and the chain length becomes shorter after purification by gel chromatography. The decrease in intramolecular and intermolecular hydrogen bonds in the molecular chains of chitosan oligosaccharides resulted in increased infrared absorption at this wave number. The C-O-C stretching vibration in the range of 1200–1050 cm^−1^ is the ether bond stretching vibration band in the pyran ring of the chitosan oligosaccharide unit structure, and the band intensity of COST is slightly higher in the figure. This may be due to the separation process affecting the ether bond, resulting in a decrease in the intensity of stretching vibrations in the separated product. Finally, the existence of such a wide peak in the figure may be caused by some water remaining in the sample.

#### 2.1.4. Determination of Deacetylation Degree

**Figure 2:**
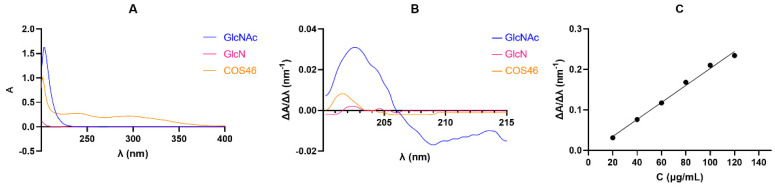
(**A**) Full sweep spectra of GlcNAc, GlcN, and COS46 at 200−400 nm wavelength; (**B**) GlcN and COS46 were mapped with the first derivative at 200–215 nm wavelength scanning; (**C**) the first derivative of GlcNAc (20.0−120.0 μg/mL) in 0.3 mol/L HCl (y = 0.002097x − 0.007467, R^2^ = 0.9924).

The results are shown in Figure 2A. Among the three, only GlcNAc had an obvious maximum absorption peak, GlcN had no obvious absorption, and COS46 showed a weak absorption peak. Then the three were scanned at a 200–215 nm wavelength and the first derivative was plotted. The results are shown in Figure 2B. The maximum absorption peak of GlcNAc and COS46 appeared near 202 nm, and the absorption of GlcNAc was significantly greater than that of COS46.

The content of GlcNAc In COS46 was calculated using the standard curve. According to the formula, the degree of deacetylation of COS46 is 97.71%, as shown in Table 2.

#### 2.1.5. Molecular Weight of COS46 and COST

The established photometric standard curve is shown in Figure S20. The linear regression equation of the standard curve is y = 0.4555x + 0.05216, and R^2^ = 0.9789.

The measurement results of COS46 and COST are shown in Table 3 and Table 4. Both OD1 and OD2 are within the linear range of the standard curve. By substituting the formula, the average molecular weight of COS46 is 628.50 and that of COST is 906.12. The average molecular weight decreased, which proved that chitosan oligosaccharides with a narrower molecular weight range were successfully obtained in the previous gel chromatography separation.

### 2.2. Oxidation Resistance of COS46 and COST

**Figure 3:**
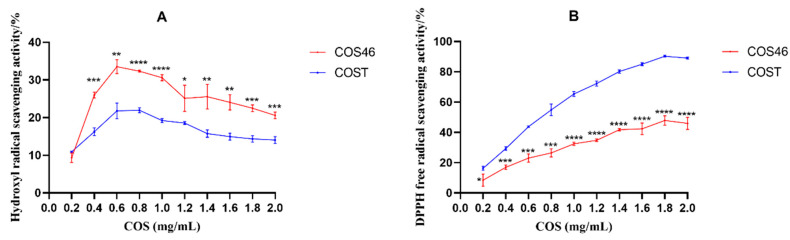
The hydroxyl radical scavenging activity (**A**); the DPPH free radical scavenging ability (**B**); * *p* < 0.05, ** *p* < 0.01, *** *p* < 0.001, **** *p* < 0.0001.

The scavenging rates of hydroxyl radicals and DPPH radicals by COS46 and COST are shown in Figure 3. For hydroxyl radical scavenging efficiency, within the experimental concentration range, except for the initial concentration of 0.2 mg/mL, the scavenging capacity of COS46 was higher than that of COST. The DPPH radical scavenging efficiency was better on COST and gradually improved with the increase in concentration. As one of the reactive oxygen species in the human body, the harm caused by the hydroxyl radical in the human body has long been recognized by the public. COS46 is more efficient than COST in reducing the formation of the hydroxyl radical, and it is more suitable as an exogenous antioxidant.

### 2.3. Effects of COS46 and COST on Hepatocyte Injury of L02

#### 2.3.1. Modeling Conditions of Hepatocyte Injury and Dose Concentration

**Figure 4:**
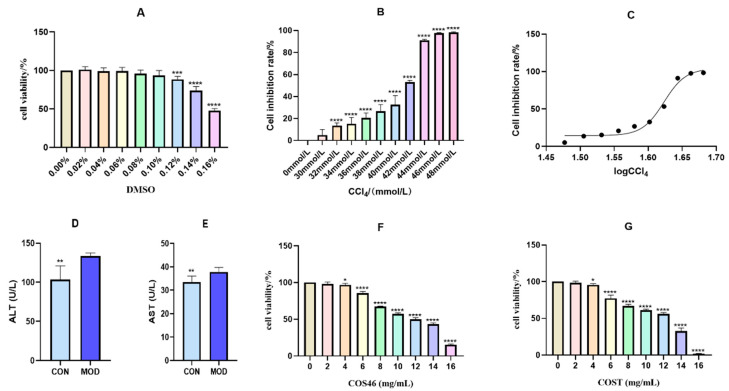
Effect of different percentage concentrations of DMSO on cell viability (**A**); inhibition rate of CCl_4_ in different concentrations (**B**); the IC_50_ curve of CCl_4_ (**C**); contents of ALT (**D**) and AST (**E**) in cell medium after 24 h treatment with CCl_4_; effect of different concentrations of COS46 (**F**) and COST (**G**) on viability of L02 cells (n = 6, mean ± SD); * *p* < 0.05, ** *p* < 0.01, *** *p* < 0.001, **** *p* < 0.0001.

As shown in Figure 4A, with the increase in the DMSO concentration, the cell viability decreased gradually. When it reached 0.12%, the cell survival rate showed a significant difference compared with no DMSO administration. Therefore, in the follow-up experiment, the proportion of DMSO selected in the modeling solution system was 0.10%.

As shown in Figure 4B,C, the cell inhibition rate increased with the increase in the CCl_4_ concentration. The modeling concentration is selected according to the principle of IC_50_, which is 42 mmol/L, and this concentration is selected as the modeling concentration.

After the modeling concentration was determined according to the IC50 curve, CCl_4_ cells attached to the wall for 24 h were further incubated for 24 h, and ALT and AST were measured in the culture medium. As shown in Figure 4D,F, ALT and AST in the MOD group were significantly increased, L02 hepatocytes were damaged, and a large number of transaminases were transferred to the culture medium. The results showed that the model of hepatocyte injury was successfully established.

As shown in Figure 4F,G. The cell survival rate decreased with the increase in the drug concentration. When the concentration of COS46 and COST reached 4 mg/mL, the cell survival rate was reduced by approximately 5% compared with the cells without drug administration, and the difference was statistically significant. When the dose was 2 mg/mL, the cell survival rate was more than 98%. Taking this as the selection standard, the high-, medium-, and low-dose concentrations of COS46 and COST were determined to be 2 mg/mL, 1 mg/mL, and 0.5 mg/mL, respectively.

#### 2.3.2. Cell Viability and Biochemical Indices

**Figure 5:**
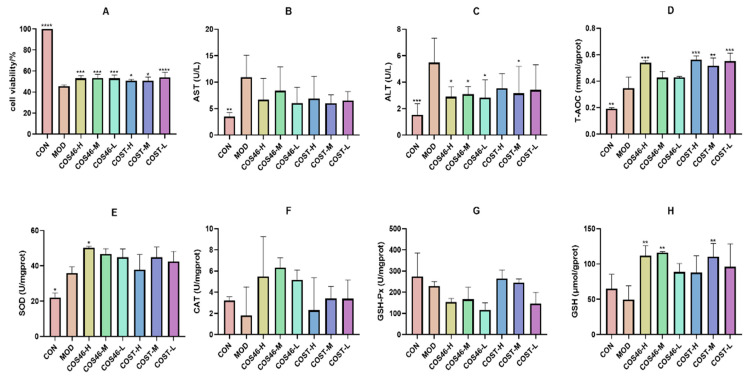
Effect of COS46 and COST on viability of L02 cells treated with CCl_4_ (**A**); effect of COS46 and COST on AST (**B**) and ALT (**C**) levels in cell medium (n = 6, mean ± SD). Effects of COS46 and COST on T-AOC (**D**), SOD (**E**), CAT (**F**), GSH-Px (**G**), and GSH (**H**) in L02 cells (n = 3, mean ± SD); * *p* < 0.05, ** *p* < 0.01, *** *p* < 0.001, **** *p* < 0.0001.

As shown in Figure 5A, the cell survival rate in the groups given COS46 and COST was significantly improved compared with the MOD group. Among them, the COS46-H,M,L had the same improvement effect on cell survival rate, and the effect was better than that of COST-H,M.

As shown in Figure 5B,C, compared with the MOD group, AST activity in each group decreased, but there was no significant difference. COS46-L and COST-M had the most obvious effect on the reduction in AST, and the effect was similar. COS46-H,M,L could reduce ALT activity, and the three doses were significantly different from the MOD group, and COS46-L was the best. Although COST-H,M,L can also affect ALT, only COST-M showed a significant difference.

The results of T-AOC, SOD, CAT, GSH-Px, and GSH levels in cells are shown in Figure 5D,H. Compared with the CON group, the T-AOC and SOD levels in the MOD group increased, and the values in each administration group were further increased than those in the MOD group. For T-AOC, COS46 showed significant differences only at high doses, while COST showed significant differences at all three doses. In terms of SOD measurement, only the COS46-H group showed a significant difference.

Compared with the CON group, the activity of CAT in the MOD group decreased, and the activity of CAT in different doses of COST and COS46 groups increased. Although there was no significant difference between the two groups, the increase in COS46 was higher than that of COST.

Compared with the CON group, the activity of GSH-Px in the MOD group decreased. After COS46 pretreatment, GSH-Px had not increased, while it had increased in COST-H,M groups, but there was no significant difference.

Compared with the CON group, the activity of GSH in the MOD group decreased, but after COS46 and COST pretreatment, the activity of GSH increased. There were significant differences between the COS46-H group and the COS46-M group, as well as between the COST-M groups.

In short, COS46 showed more obvious improvement in SOD, CAT, and GSH, but COST has a greater influence on T-AOC and the GSH-Px index. Both COS46 and COST can reduce the oxidative damage of CCl_4_ to hepatocytes. Combined with the experimental results, COS46 has a more favorable improvement effect.

### 2.4. Effects of COS46 and COST on Mice with Liver Injury

#### 2.4.1. Effects of COS46 and COST on Liver Appearance, Liver Index, and Serum AST and ALT

**Figure 6:**
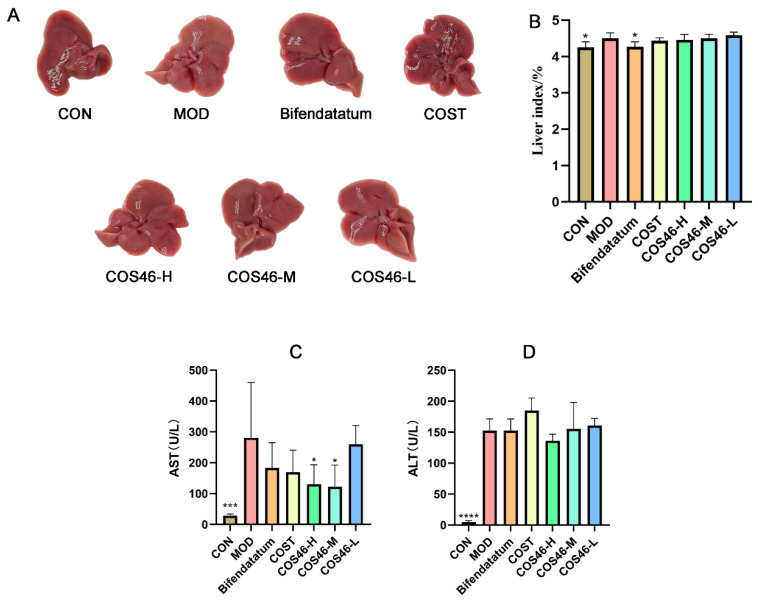
Appearance of mouse liver (**A**); liver index (**B**); AST (**C**) and ALT (**D**) in serum (n = 6, mean ± SD); * *p* < 0.05, *** *p* < 0.001, **** *p* < 0.0001.

As shown in Figure 6A, the livers of mice in the CON group were ruddy in appearance and exquisite in texture, and no granular feeling was observed. The livers of the MOD group showed obvious yellow granules, a yellow surface, and decreased elasticity. Yellow particles were also observed on the liver surfaces of the positive drug group, but no yellowing or redness was restored compared with the MOD group. CCl_4_-caused liver lesions were improved in all groups given chitosan oligosaccharides. As the dosage of COS46 increased, the effect became more obvious, and the particle sensation was lighter than that of COST.

As shown in Figure 6B, the liver index of the CON group was significantly lower than that of the MOD group, indicating that liver swelling and other conditions occurred after the administration of CCl_4_. The liver index of COS46 dose groups decreased in a dose-dependent manner, but there was no significant difference compared with the MOD group.

As shown in Figure 6C,D, compared with the CON group, AST and ALT indexes in the MOD group significantly increased, indicating that CCl_4_ caused liver cell damage. After pretreatment for 7 days, the serum AST value of each group decreased, and the COS46-H group and the COS46-M group showed significant differences. The ALT index did not decrease significantly, but the COS46 group showed dose dependency.

#### 2.4.2. Mouse Liver Biochemical Indexes and HE Staining

**Figure 7:**
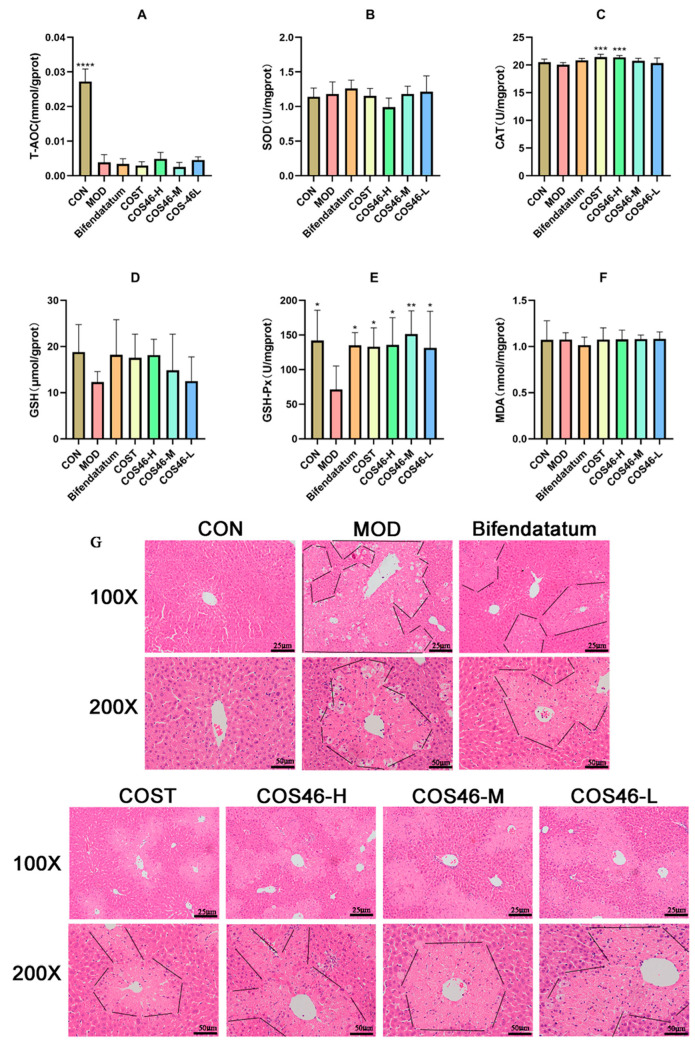
The antioxidant–oxidative index of liver (n = 6, mean ± SD), T-AOC (**A**), SOD (**B**), CAT (**C**), GSH (**D**), GSH-Px (**E**), and MDA (**F**); liver HE staining (**G**); * *p* < 0.05, ** *p* <0.01, *** *p* < 0.001, **** *p* < 0.0001. Black indicates the outline of the damaged part.

Figure 7A–F shows the detection results of antioxidant enzyme activity and oxidative damage indexes in the livers of mice in each group. Compared with the CON group, the total antioxidant capacity of the MOD group significantly decreased, while T-AOC did not significantly increase in all administration groups, but the values of COS46-H and COS46-L groups increased slightly. There was no significant difference in SOD levels among all groups.

Compared with the CON group, CAT enzyme activity in the MOD group decreased slightly, but there was no significant difference. In each administration group, the enzyme activity of the COST group and the COS46-H group increased compared with that of the MOD group and showed a significant difference. Meanwhile, COS46 showed a dose-dependent increase in CAT enzyme activity.

The content of GSH in the MOD group was much lower than that in the CON group, which showed no significant difference. In each administration group, the GSH content in the liver increased, and COS46 increased the GSH content in a dose-dependent manner, but there was no significant difference. The activity of the GSH-Px enzyme in the MOD group significantly decreased, while the activity of GSH-Px significantly increased in all administration groups, and COS46-M showed the best effect. In short, COS46 is better than COST in improving GSH and GSH-Px.

The levels of MDA in the CON group and the MOD group were close to each other, but there was no change trend in MDA content in each administration group.

The HE staining results of mouse livers in each group are shown in Figure 7G. The liver structure of the CON group was normal, and cells were arranged uniformly and in order, with the central vein as the center in a single radial arrangement. In the MOD group, there were obvious abnormalities, such as vacuolar degeneration, necrosis, nuclear ruptures, and cytoplasmic staining of hepatocytes. After 7 days of bifendatatum pretreatment, liver pathological sections showed some improvement in liver abnormalities and no obvious vacuole-like degeneration, and the degree of cytoplasmic redness was reduced. The COST group and the COS46-H group also had the effect of alleviating liver injury and the degree of the two groups was like that of the positive-drug group under visual observation, and both were slightly weaker than the positive-drug group. Similarly, the effects of COS46-M and COS46-L groups on liver injury were similar, with no significant difference, but compared with the MOD group, they could still improve the damage caused by CCl_4_.

## 3. Materials and Methods

### 3.1. Materials

COST was purchased from AK Biotech Co., Ltd. (Chengdu, China) (detailed parameters can be found in the certificate of analysis in supplementary data).

The COS4 standard, COS5 standard, COS6 standard, and chitosan oligosaccharides mixed standard (DP1-7) were purchased from Huizhou Changlong Biotechnology Co., Ltd. (Guangdong, China).

The biochemical index kits used in this paper were purchased from Nanjing Jiancheng Bioengineering Institute.

The chemicals used are commercially available products.

### 3.2. COST Component Analysis and Purification of COS46

#### 3.2.1. COST Component Analysis and DP4-6 Component Content Determination

(1) Qualitative analysis of COST components by TLC thin layer

COST and chitosan oligosaccharides mixed standards (dp1-7) were used to prepare the solution with the appropriate concentration. Plate: Merck high-efficiency silicone plate (1.05553). Development height: 9.5 cm. Developer system: isopropanol: ammonia: pure water = 15:7.5:1. After unfolding, the plate was dried, soaked in anisaldehyde sulfate colorant for 2 min, dried, and heated in an oven at 110 °C for 10 min to develop color.

(2) The content of DP4-6 components in COST was analyzed by LC-MS

The COST sample and chitosan oligosaccharides standard were weighed and a series of solutions were prepared as follows:

The COS4 standard was weighed as 2.80 mg, dissolved in (acetonitrile: water = 3:7) to make a 1.40 mg/mL solution, and diluted to 0.7, 0.35, 0.175, and 0.0875 mg/mL, respectively. 

The COS5 standard was weighed as 2.10 mg, dissolved in (acetonitrile: water = 3:7) to make a 1.40 mg/mL solution, and diluted to 0.35, 0.175, 0.0875, and 0.04375 mg/mL, respectively.

The COS6 standard was weighed as 1.80 mg, dissolved in (acetonitrile: water = 3:7) to make a 1.40 mg/mL solution, and diluted to 0.45, 0.225, 0.1125, and 0.05625 mg/mL, respectively.

The COST standard was weighed as 3.4 mg and dissolved in (acetonitrile: water = 3:7) to make a 3.44 mg/mL solution.

Ten microliters of each solution were extracted for analysis and the standard curve was established. 

Table 5 lists the experimental parameters of LC-MS.

#### 3.2.2. Isolation and Identification of COS46

(1) GPC

The Sephadex G-15 was used as column packing, which was fully swelled in a 90 ℃ water bath and cleaned and had the gas removed, and the chromatographic column was installed by the wet method.

The G15 column was used at a flow rate of 3 mL/h to separate. Then it was collected and lyophilized. The collection frequency was once every 20 min until the reception was completed. This part of the operation is based on TLC operation described in Section 3.2.1 (1). The effluents containing chitosan oligosaccharides with DP = 4–6 were selected for consolidation and freeze-dried, and the yield was calculated according to the mass.

(2) LC-MS

The experimental conditions of LC-MS were the same as in Section 3.2.1 (2).

First, 1.82 mg COS46 was weighed and dissolved (acetonitrile: pure water = 3:7) into a 1.82 mg/mL solution, and 10 μL was injected for analysis.

(3) FT-IR

The COS46 and COST samples were mixed with dry potassium bromide, and then placed in an agate mortar. After grinding and mixing, the tablets were pressed on an oil press. The baseline was calibrated with dry potassium bromide. The scanning range was 400–4000 cm^−1^, the resolution was 4 cm^−1^, and the number of scans was 16. The infrared spectra of COS46 and COST were obtained.

#### 3.2.3. Determination of Deacetylation Degree

The deacetylation degree of COS46 was determined by first-derivative ultraviolet spectrophotometry established in the laboratory [43].

(1) Determination of maximum absorption wavelength

GlcNAc (N-acetylglucosamine), GlcN (aminoglucose), and COS46 solutions were prepared in a 0.3 mol/L HCl solution. The scanning wavelength range was 200–400 nm, the slit width was 2 nm, the scanning speed was 20 nm/min, the time constant was 4S, and the recording speed was 10 cm/min. The optical path of the quartz cuvette is 1 cm. Then, under spectral scanning in the range of 200–215 nm, the absorbance of the solution was recorded, and the absorbance was converted into first-order differential calculation.

(2) First-derivative ultraviolet spectrophotometry was established

GlcNAc standard solutions with different concentrations were prepared in a HCl solution. The absorbance of each standard solution was recorded at the main wavelength of 204 nm and the baseline wavelength of 202 nm, and ΔAΔλ (ΔA = A204 nm–A202 nm, Δλ = 2 nm) was calculated. The concentration of the standard solution was the abscissa, and ΔAΔλ was the ordinate to establish the standard curve.

(3) Determination of deacetylation degree of COS46

$$DD\left(\%\right)=\frac{C_{46}-C_{GlcNAc}}{C_{46}-\frac{42}{203}C_{GlcNAc}}\times 100$$

*C46* is the concentration of COS46 (μg/mL); *C_GlcNAc_* is the concentration of the N-acetylglucosamine unit in the sample, which is obtained according to the standard curve (μg/mL); 203 is the molecular weight of the *GlcNAc* fragment in COS46, calculated according to C_8_H_13_NO_5_; 42 is the relative molecular weight difference between *GlcNAc* and GlcN fragments.

#### 3.2.4. Determination of Number Average Molecular Weight of COST and COS46

A 1 mg/mL glucosamine standard solution was prepared as solutions of different concentrations and added to different glass tubes. Each glass tube was filled with distilled water to 1 mL, 1 mL of the DNS reagent was added, it was placed in a boiling water bath for 5 min, 8 mL of distilled water was added, and 200 μL was removed and placed in a 96-well plate. *OD1* (optical density) was measured at 520 nm by a microplate reader.

Then, 1 mg/mL COST and COS46 solutions were prepared. The remaining operations were the same as above. The *OD1* value was determined at 520 nm with an enzyme-labeled instrument.

Next, we took 1 mL of 1 mg/mL COST and COS46, added 3 mL of 6 mol/L HCl, bathed it in boiling water for 2 h, neutralized the mixture with 6 mol/L NaOH, and diluted the solution to 10 mL with water. We then took 1 mL of diluted hydrolysate, added 1 mL of the DNS reagent, bathed the mixture in boiling water for 5 min, and added 8 mL of distilled water after cooling. Finally, we removed 200 μL and measured the *OD2* value with an enzyme-labeled instrument at 520 nm.

If both *OD1* and *OD2* are in the range of the scalar curve, the average degree of polymerization of the sample is:

$$n=10\times \frac{OD1}{OD2}$$

The average relative molecular mass of the sample is:

$$M_{W}=n\times 179-(n-1)\times 18$$

### 3.3. Determination of Oxidation Resistance of COS46 and COST

#### 3.3.1. Determination of Hydroxyl Radical Scavenging Ability

In each test tube, 1 mL of COS46 and COST (in concentrations of 0.2, 0.4, 0.6, 0.8, 1.0, 1.2, and 1.4 mg/mL) was added, followed by the sequential addition of 1 mL of 6 mmol/L FeSO_4_, 2 mL of 6 mmol/L H_2_O_2_, and mixing. The mixture was then left in the dark at room temperature for 10 min. Subsequently, 1 mL of a 6 mmol/L salicylic acid-ethanol solution was added, and after mixing, the reaction was held in the dark at 37 °C for 60 min in a water bath. After cooling to room temperature, the absorbance was measured at 517 nm, yielding the absorbance value *A1*. For the blank group, an equivalent amount of water served as the substitute for the sample, yielding the absorbance value *A0.* Each group underwent three parallel measurements. The hydroxyl radical scavenging rate was then calculated using the formula below:

$$Hydroxyl\;radical\;scavenging\;activity\left(\%\right)=\frac{A_{0}-A_{1}}{A_{0}}\times 100\%$$

#### 3.3.2. Determination of DPPH Free Radical Scavenging Ability

First, we added 2 mL of the 0.1 mmol/L DPPH (1,1-Diphenyl-2-picrylhydrazyl radical)-EtOH solution and 2 mL of different concentrations of COS46 and COST (0.2, 0.4, 0.6, 0.8, 1.0, 1.2, 1.4, 1.6, 1.8, and 2.0 mg/mL) to each test tube, mixed well, and reacted the solution in a dark water bath at 37 °C for 60 min. After cooling to room temperature, we measured the absorbance at 517 and recorded it as *A1*. The control group used the same amount of absolute ethanol instead of the DPPH-EtOH solution, which was recorded as *A2*, and the blank group used the same amount of distilled water instead of the sample to be tested. We then calculated the DPPH radical scavenging rate according to the following formula:

$$DPPH\;free\;radical\;scavenging\;activity\left(\%\right)=\left(1-\frac{A_{1}-A_{2}}{A_{0}}\right)\times 100\%$$

### 3.4. Cell Experiment

#### 3.4.1. L02 Cell Culture

L02 cells were cultivated in a constant-temperature and -humidity environment at 37 °C and 5% CO_2_ in a complete medium containing RPMI-1640, fetal bovine serum (9:1, *v*/*v*), streptomycin (100 μg/mL), and penicillin (100 U/mL), with a 1% double-antibody ratio. The culture medium was replaced every two days, and the experiment was initiated when the cell fusion rate reached 80%.

#### 3.4.2. Establishment of Liver Injury Model Induced by CCl4 (Determination of DMSO and CCl4 Concentrations)

First, 0.02%, 0.04%, 0.06%, 0.08%, 0.10%, 0.12%, 0.14%, 0.16%, and 0.18% DMSO were used to treat cells.

Next, 10, 20, 30, 40, 50, 60, 70, 80, and 90 mmol/L CCl_4_ were used to treat cells. CCK-8 was used to determine the cell survival rate, and IC50 was calculated to determine the modeling concentration.

#### 3.4.3. The Concentrations of COS46 and COST

First, 2, 4, 6, 8, 10, 12, 14, and 16 mg/mL COS46 and COST were given to cells, respectively, and then they were cultured for 24 h. The CCK-8 method was used to determine the cell inhibition rate and the dosage of COS46 and COST.

#### 3.4.4. Effects of COS46 and COST on Survival Rate of L02 Cells Treated with CCl_4_

L02 cells at the logarithmic growth stage were inoculated into 96-well culture plates with 2.0 × 10^4^ cells per well. The CON group, MOD group, COS46-H/M/L group, and COST-H/M/L group were set up and cultured for 24 h. The administration group was added with different concentrations of drugs and continued to culture for 24 h. In addition to CON, each group was added with the modeling concentration of CCl_4_ and continued to be treated for 24 h. CCK-8 measured the cell survival rate.

#### 3.4.5. Culture Medium and Antioxidant and Oxidation Indices Detection

As described in Section 3.4.4, the supernatant of the medium was collected and the contents of ALT and AST were measured.

L02 cells at the logarithmic growth stage were inoculated into 6-well culture plates with 4 × 10^5^ cells per well. The cells were then lysed, and the lysate was collected to determine the levels of T-AOC (Total antioxidant capacity), SOD (Superoxide dismutase), CAT (Catalase), GSH-Px (Glutathione peroxidase), and GSH (glutathione.).

### 3.5. Animal Experimental

#### 3.5.1. Animal

Seventy SPF male C57BL/6J mice, 18–22 g, were purchased from Guangdong Sijia Jingda Biological Co., LTD., Production License No.: SCXK (Guangdong, China) 2020-0052; Reared in Animal Center of Guangdong Pharmaceutical University, License No. SYXK (Guangdong, China) 2022-0125. The feeding conditions of the animal experiment were 24.0 ± 2.0 °C, relative humidity of 54–65%, air change times > 15 times /h, and alternating light and dark for 12 h. The experimental animals are kept in the pathogen-free laboratory of the Experimental Animal Center of Guangdong Pharmaceutical University. This experiment was approved by the Experimental Animal Ethics Committee of Guangdong Pharmaceutical University and strictly followed the requirements of the Guidelines for Ethical Review of Experimental Animal Welfare (GB/T35892-2018) to fully protect the welfare of experimental animals.

#### 3.5.2. Administration and Establishment of Acute Liver Injury Model

Seventy male C57BL/6J mice were randomly divided into 7 groups after 1 week of adaptive feeding. They were the blank control group (CON), model control group (MOD), positive drug control group (Bifendatatum), COST administration group (COST), COS46 high-dose group (COS46-H), COS46 medium-dose group (COS46-M), and COS46 low-dose group (COS46-L). There were 10 mice in each group, and they all had a free diet.

The COST dose was 250 mg/kg, and the COS46 high, medium and low doses were 500 mg/kg, 250 mg/kg, and 125 mg/kg, respectively. The blank control group and the model control group were intragastric with distilled water instead, and intragastric administration continued for 7 days. After the end of the 7th day, the liver injury model was induced by a one-time gavage of a 5 mL/kg 1% CCl_4_ oil solution in each dose group and the model control group and the corresponding volume of olive oil was given to the blank control group, and then the tested animals were fasted for 16 h. The mice were then anesthetized by an intraperitoneal injection of a 60 mg/kg BW sodium pentobarbital solution, and blood was taken from the orbit. The animals were sacrificed by cervical dislocation after blood collection. The appropriate amount of liver was taken for embedding, fixed with 4%PFA for 24 h, and the remaining liver was stored at −80 °C for use.

(1) Liver index

Mouse body weight and liver weight were weighed, and the liver index was calculated.

$$Liver\;index\;\left(\%\right)=\frac{liver\;weight\;(g)}{body\;weight\;(g)}$$

(2) Biochemical index

Serum: ALT and AST

Liver: T-AOC, SOD, MDA, CAT, GSH, GSH-Px

The above indexes were measured by the Nanjing Jiancheng Kit.

(3) Liver HE staining

The liver was embedded in paraffin wax and cut into 4 μm pathological sections for HE staining (Leagene Biotechnology, Beijing, China, DH0006).

### 3.6. Statistical Analysis

The data obtained in this experiment were analyzed and mapped by GraphPad Prism 9.0, and the result of the data was expressed as the mean ± SD. The data significance analysis was conducted using One-Way ANOVA for multi-group data and T-tests for the data significance analysis between the two groups. *p* < 0.05 indicated statistical significance.

## 4. Discussions and Conclusions

Traditionally, chitosan oligosaccharides are a mixture prepared from chitin or chitosan. When using such chitosan oligosaccharides to explore their biological activity, the repeatability of the experiment is poor, and the results may be divided, so the study of separating chitosan oligosaccharides will definitely become a trend in the future. In addition, because chitosan oligosaccharides with different polymerization degrees have different biological activities [44], a detailed study on chitosan oligosaccharides with different polymerization degrees is conducive to the safer and more reasonable use of chitosan oligosaccharides and will further expand the application scope of chitosan oligosaccharides.

In this study, COS46 contains a tetramer, a pentamer, and a hexamer, and it is regarded as a whole as the research object, and the effect of each component is not analyzed in detail. However, according to the results provided by TLC, G-15 was selected as chromatographic column packing, according to its retention effect on chitosan oligosaccharides with different molecular weights. Reevaluating the height, diameter, and flow rate of the packing, it is possible to further separate the three components (COS4, COS5, and COS6) of COS46. 

During the antioxidant activity test, it was observed that chito-oligosaccharides, both before and after separation, demonstrated a superior scavenging ability of free radicals. Hydroxyl free radicals, which are considered harmful to the human body, can form within the body. COS46 was found to be more effective in scavenging hydroxyl free radicals. When foreign substances stimulate the human body, hydrogen peroxide is produced. This then reacts with Fe^2+^ to generate reactive oxygen through the Fenton reaction. It is speculated that COS46 can chelate with Fe2+ more effectively to prevent their reaction [45,46]. Therefore, COS46 may have more potential as an antioxidant.

After using CCl_4_ to establish liver injury modeling, this experiment only explored the effects of COST and COS46 on L02 hepatocyte injury, antioxidant and oxidative effects, and effects on the Keap-1/Nrf2/HO-1 pathway in L02 hepatocytes. The change in related indexes after drug administration was not discussed further. Based on the experiment, it was found that SOD and CAT enzyme activities were higher in the cell administration groups compared to the CON group. This, combined with the existing research, suggests that COS46 and COST treatment may activate the signaling pathway, increase the expression of related antioxidant proteins, and improve the activity of SOD, CAT, and other antioxidant enzymes to some extent. When cells are exposed to toxic substances, they can quickly develop protective mechanisms to reduce damage and prevent cell death. The MOD group showed higher T-AOC and SOD indexes compared to the CON group. This may be due to the insufficient concentration of CCl_4_ used in the liver toxicity model to cause cell death in all cells, and the surviving cells were able to respond positively and significantly improve their enzyme activity, allowing them to survive in the presence of CCl_4_.

Many studies have verified the protective effect of chitosan oligosaccharides on the liver [47,48,49]. However, their administration time is usually more than 4 weeks, and studies on the short-term effect of chitosan oligosaccharides are very rare. Therefore, in animal experiments, we chose to verify the protective effect of administration for one week on acute liver injury.

After seven days of pretreatment and gavage with CCl_4_, the MOD group of mice exhibited liver damage, including an increased liver index, apparent granulation, and elevated serum AST and ALT levels. Each administration group was able to alleviate the injury’s deterioration. Additionally, it was observed that administration could reverse the effects of CCl_4_, leading to a reduction in liver GSH and Gsh-Px content and resistance to oxidative damage. However, the detection of related oxidation indexes yielded slightly different trends between the results of the cell experiment and the animal experiment. It is speculated that there may be differences between human hepatocytes and mouse cells in vitro, resulting in varying sensitivities to drugs and a slight deviation in the experimental results of the two cell types. It is also possible that the administration time in animal experiments is insufficient, which shows that chitosan oligosaccharides can only be used as a dietary supplement for a long time, and its short-term liver protection effect is not significant enough.

In summary, this paper demonstrates that the process of obtaining COS46 through GPC separation and purification has good process stability and high product purity. This method overcomes the current difficulties in preparing low-molecular-weight chitosan oligosaccharides to some extent. Simultaneously, COS46 and COST can act as exogenous antioxidants and improve the body’s antioxidant levels. This can effectively prevent damage to human hepatocytes and mouse livers caused by CCl_4_ to a certain extent. Low-molecular-weight COS46 is superior to COST in some aspects of antioxidation.

## Figures and Tables

**Table 1:** Yield of COS46 obtained from GPC separation and purification.

Mass_COST_ (g)	Mass_COS46_ (g)	Yield (%)	Average Yield (%)
0.3059	0.0891	29.13	30.39 ± 1.57
0.3343	0.1002	29.97	
0.3290	0.1055	28.72	
0.3289	0.1055	32.08	
0.3228	0.1035	32.06	

**Table 2:** Degrees of deacetylation of COS46.

Sample	Concentration cos_46_ (μg/mL)	Deacetylation Degree (DD)%	Average DD (%)
COS46-1	705.3	97.23	97.71
COS46-2	705.3	97.33	
COS46-3	705.3	98.58	

**Table 3:** Average molecular weight of COS46.

OD1-COS46		OD2-COS46		DP	Average Molecular Weight
Absorbance	Average	Absorbance	Average		
0.2706	0.2711	0.1021	0.1028	3.79	628.50
0.2676		0.1040			
0.2752		0.1023			

**Table 4:** Average molecular weight of COST.

OD1-COST		OD2-COST		DP	Average Molecular Weight
Absorbance	Average	Absorbance	Average		
0.2342	0.2108	0.1131	0.1135	5.38	906.12
0.2536		0.1175			
0.1445		0.1098			

**Table 5:** Experimental conditions of liquid chromatography–mass spectrometry (LC-MS).

	Item	Condition
LC	Column	GL Sciences Inertsil NH_2_ 3.0 mm × 150 mm, 5 µm
	T	35 °C
	μ	0.6 mL/min
	Mobile phase	CH₃CN:0.3%NH_3_∙H_2_O = 65:35
MS	Spray Voltage	3200 V
	Capillary Temperature	270.00 °C
	Sheath Gas	40.00 Arb
	Aux Gas	8.00 Arb
	Max Spray Current	100.00 µA
	Probe Heater Temp.	250.00 °C
	Ion Source	ESI + ms

## Data Availability

The data from the present study are available in the article.

## Supplementary Materials

The following supporting information can be downloaded at: https://www.mdpi.com/article/10.3390/md22030128/s1, Figure S1: 0.0875 mg/mL COS4 (A), 0.04375 mg/mL COS5 (B), 0.05625 mg/mL COS6 (C) chromatograms of standard products; Figure S2: 0.175 mg/mL COS4 (A), 0.0875 mg/mL COS5 (B), 0.1125 mg/mL COS6 (C) chromatograms of standard products; Figure S3: 0.350 mg/mL COS4 (A), 0.175 mg/mL COS5 (B), 0.225 mg/mL COS6 (C) chromatograms of standard products; Figure S4: 0.700 mg/mL COS4 (A), 0.350 mg/mL COS5 (B), 0.450 mg/mL COS6 (C) chromatograms of standard products; Figure S5: 1.400 mg/mL COS4 (A), 0.700 mg/mL COS5 (B), 0.900 mg/mL COS6 (C) chromatograms of standard products; Figure S6: Liquid phase diagram of 1.820 mg/mL COS46; Figure S7: Liquid phase diagram of 3.440 mg/mL COST; Figure S8: Mass spectrum of COS46 when RT = 3.60 min; Figure S9: Mass spectrum of COS46 when RT = 4.78 min; Figure S10: Mass spectrum of COS46 when RT = 6.44 min; Figure S11: Mass spectrum of COS46 when RT = 8.83 min; Figure S12: Mass spectrum of COS46 when RT = 12.65 min; Figure S13: Mass spectrum of COS46 when RT=18.10min; Figure S14: Mass spectrum of COST when RT = 3.55 min; Figure S15: Mass spectrum of COST when RT = 4.83min; Figure S16: Mass spectrum of COST when RT = 6.56 min; Figure S17: Mass spectrum of COST when RT = 9.01 min; Figure S18: Mass spectrum of COST when RT = 12.98 min; Figure S19: Mass spectrum of COST when RT = 18.67 min; Figure S20: Standard curve of photometric method; Table S1: Standard curves of retention time (RT) and concentrations of COS4, COS5 and COS6; Table S2: Liquid phase data of COS46 and COST target components.

## Abbreviations

COS	chitosan oligosaccharides
COST	chitosan oligosaccharides (Mw ≤ 1000)
COS46	chitosan oligosaccharides with degree of 4–6 polymerization
COS4	chitotetraose
COS5	chitopentaose
COS6	chitohexaose
DD	degree of deacetylation
DP	degree of polymerization
GlcNAc	acetylglucosamine
GlcN	aminoglucose
