# Supplementary material for: Isolation and Purification of Chitosan Oligosaccharides (Mw ≤ 1000) and Their Protective Effect on Acute Liver Injury Caused by CCl4
Source: Mar Drugs. 2024 Mar 8;22(3):128. doi: 10.3390/md22030128 (PMC10971719; doi:10.3390/md22030128)
Supplement: Supplementary file 1 [file marinedrugs-22-00128-s001.zip › marinedrugs-2820153-supplementary.pdf]

Supplementary data

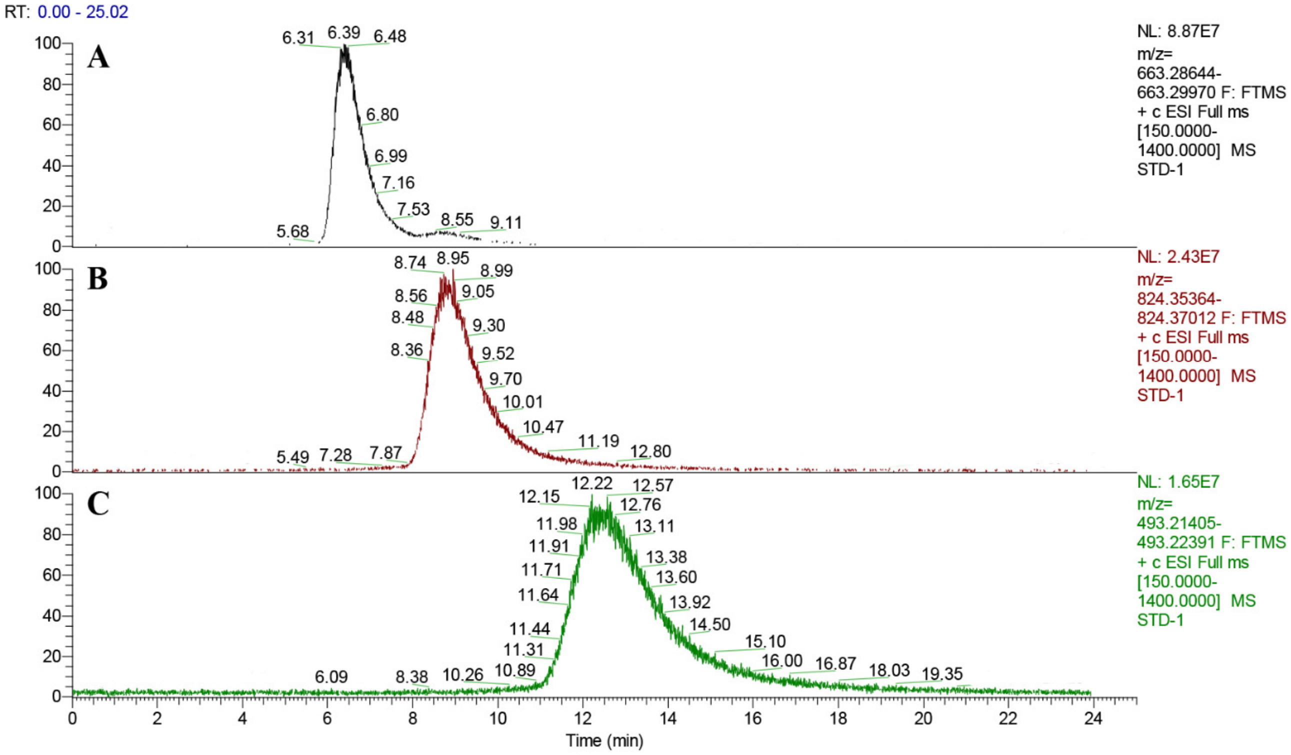

Figure S1 0.0875mg/mL COS4 (A), 0.04375 mg/mL COS5 (B), 0.05625 mg/mL COS6 (C) chromatograms of standard products

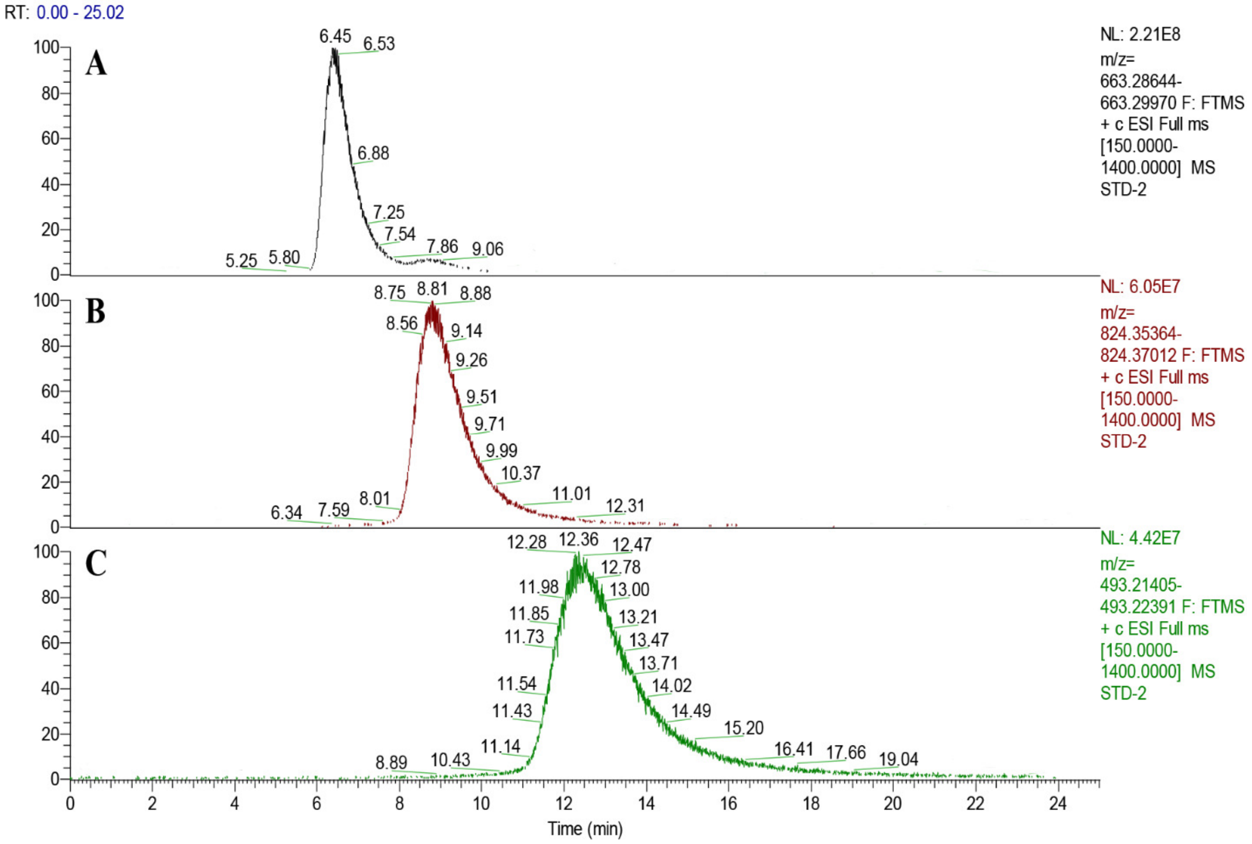

Figure S2 0.175mg/mL COS4 (A), 0.0875mg/mL COS5 (B), 0.1125mg/mL COS6 (C) chromatograms of standard products

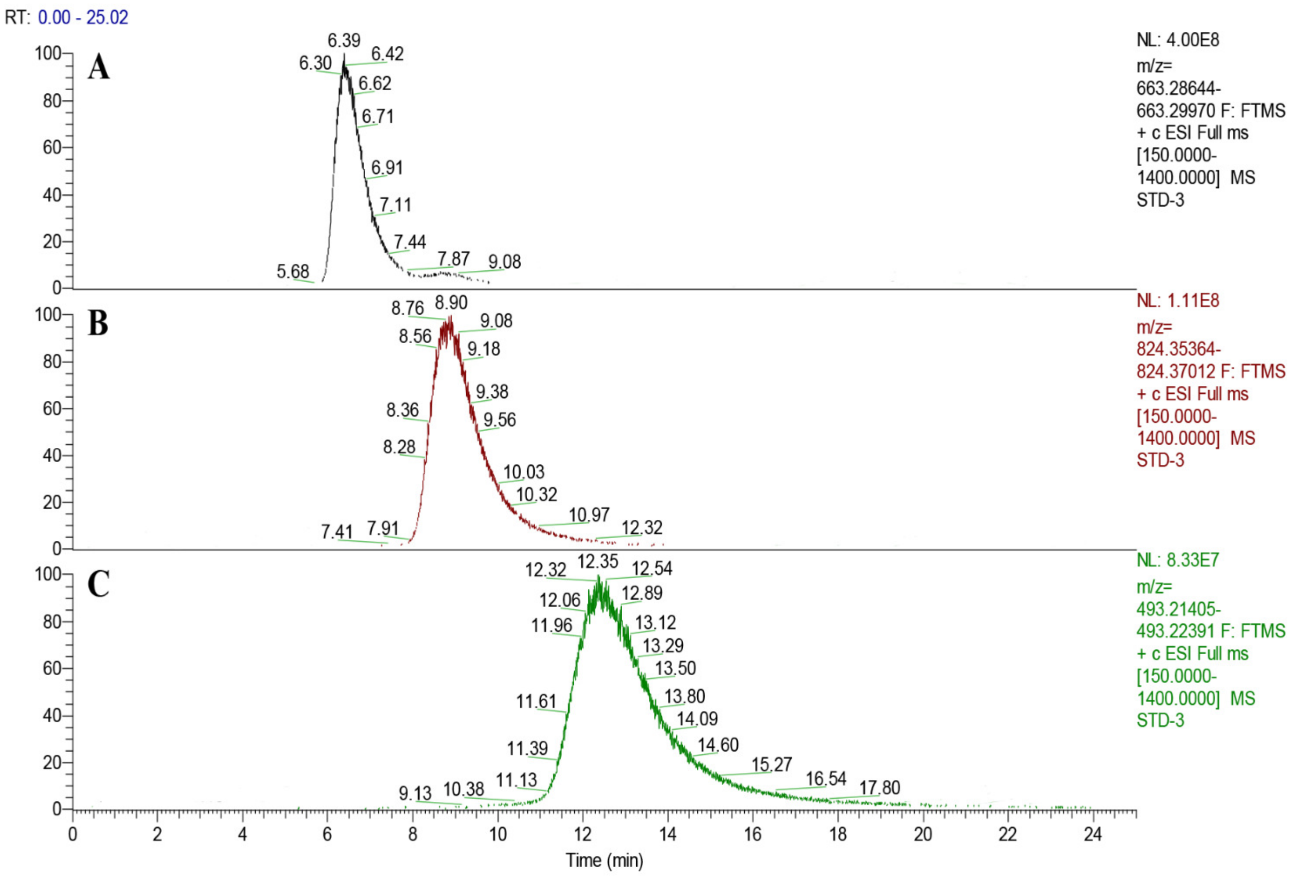

Figure S3 0.350mg/mL COS4 (A), 0.175mg/mL COS5 (B), 0.225mg/mL COS6 (C) chromatograms of standard products

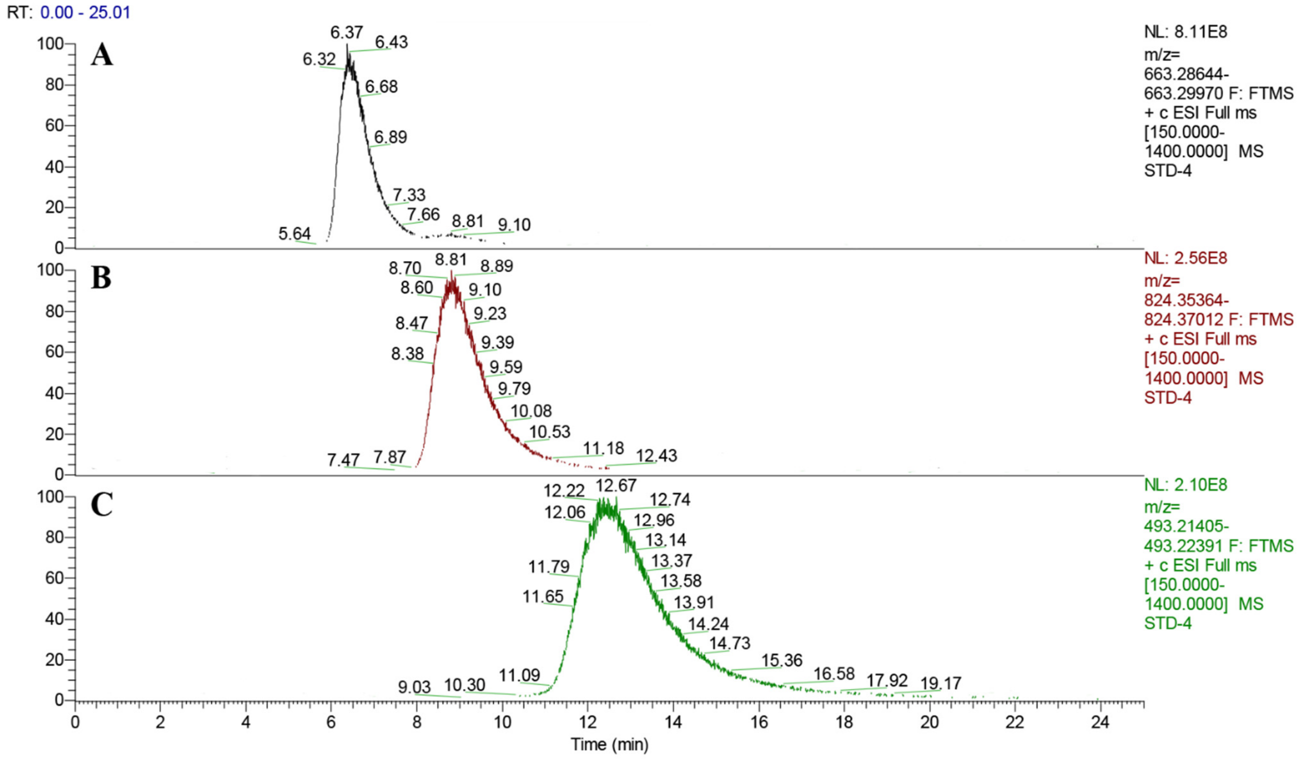

Figure S4 0.700mg/mL COS4 (A), 0.350mg/mL COS5 (B), 0.450mg/mL COS6 (C) chromatograms of standard products

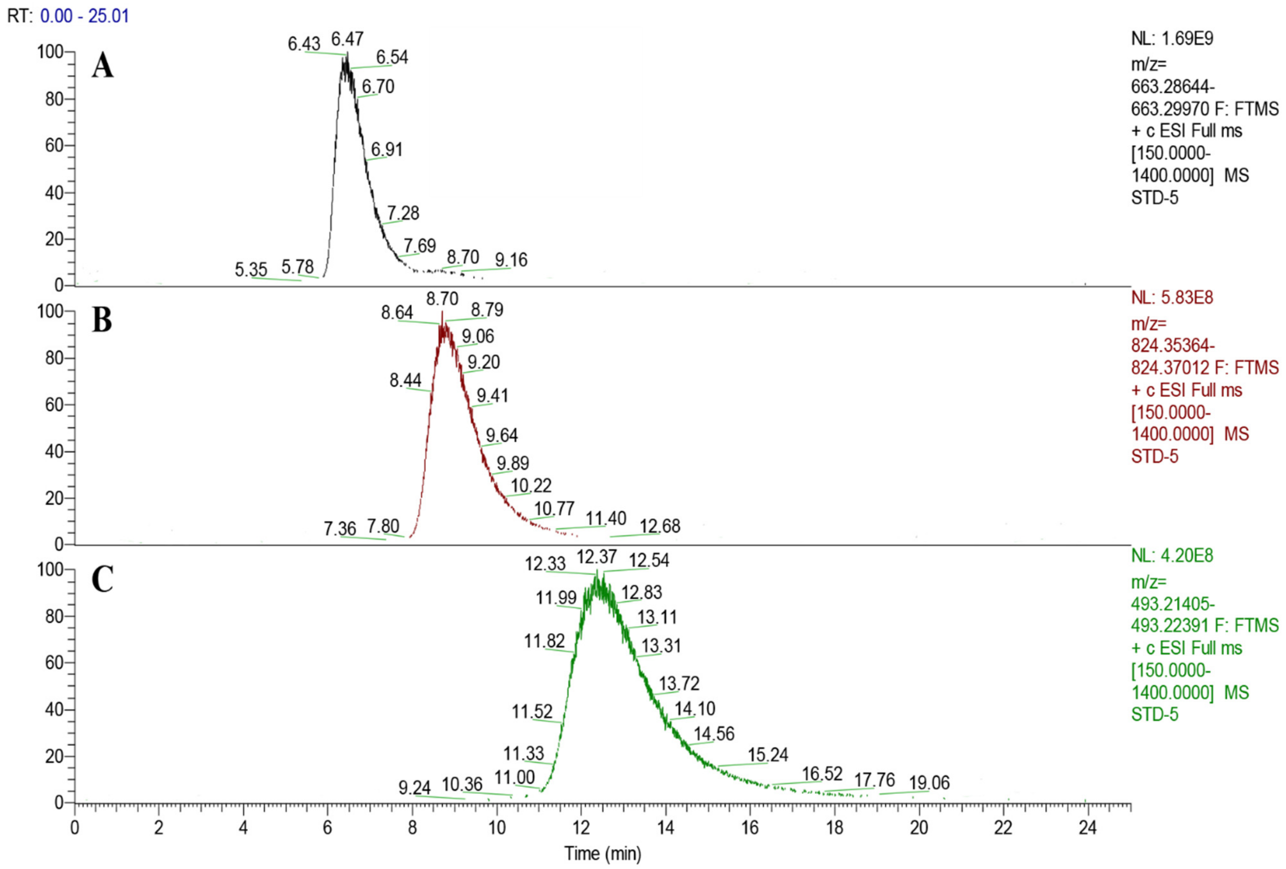

Figure S5 1.400mg/mL COS4 (A), 0.700mg/mL COS5 (B), 0.900mg/mL COS6 (C) chromatograms of standard products

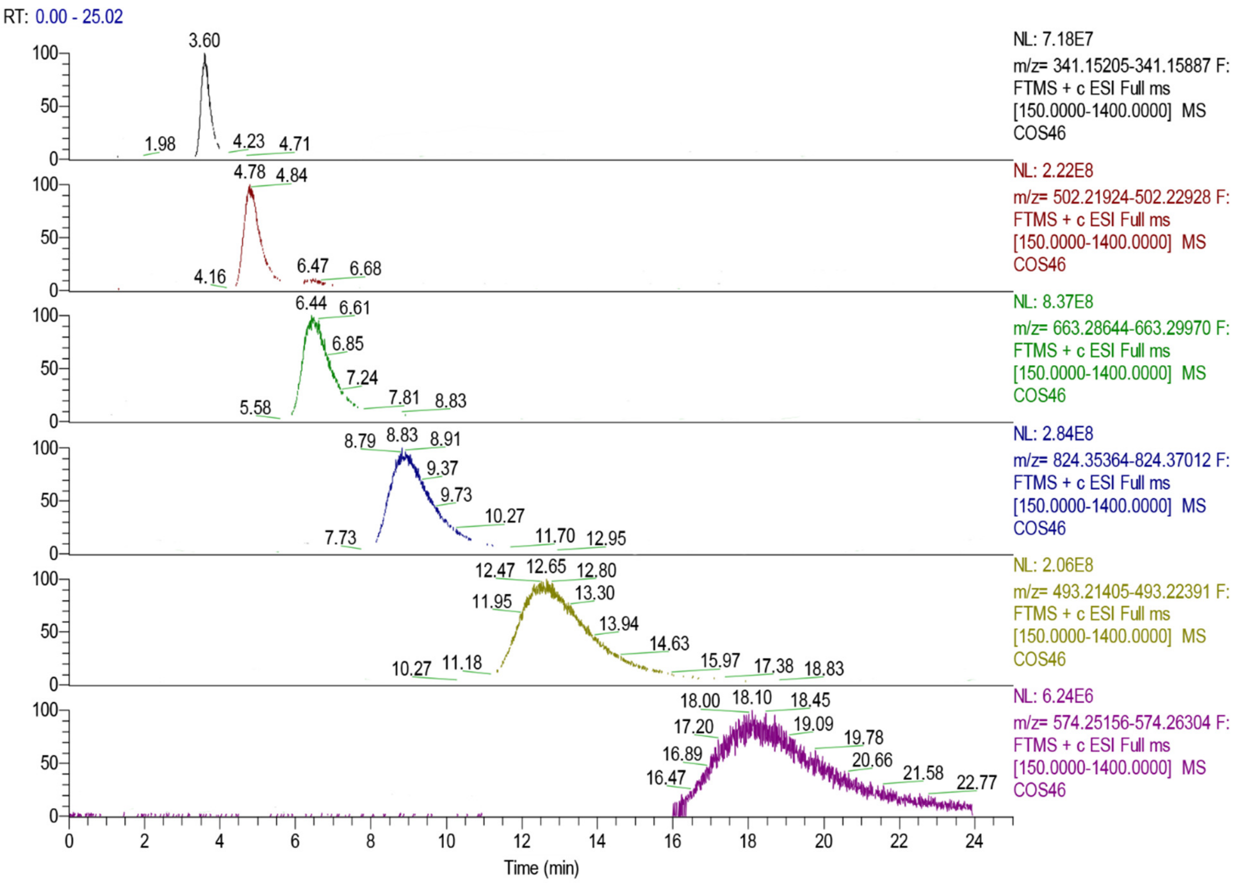

Figure S6 Liquid phase diagram of 1.820mg/mL COS46

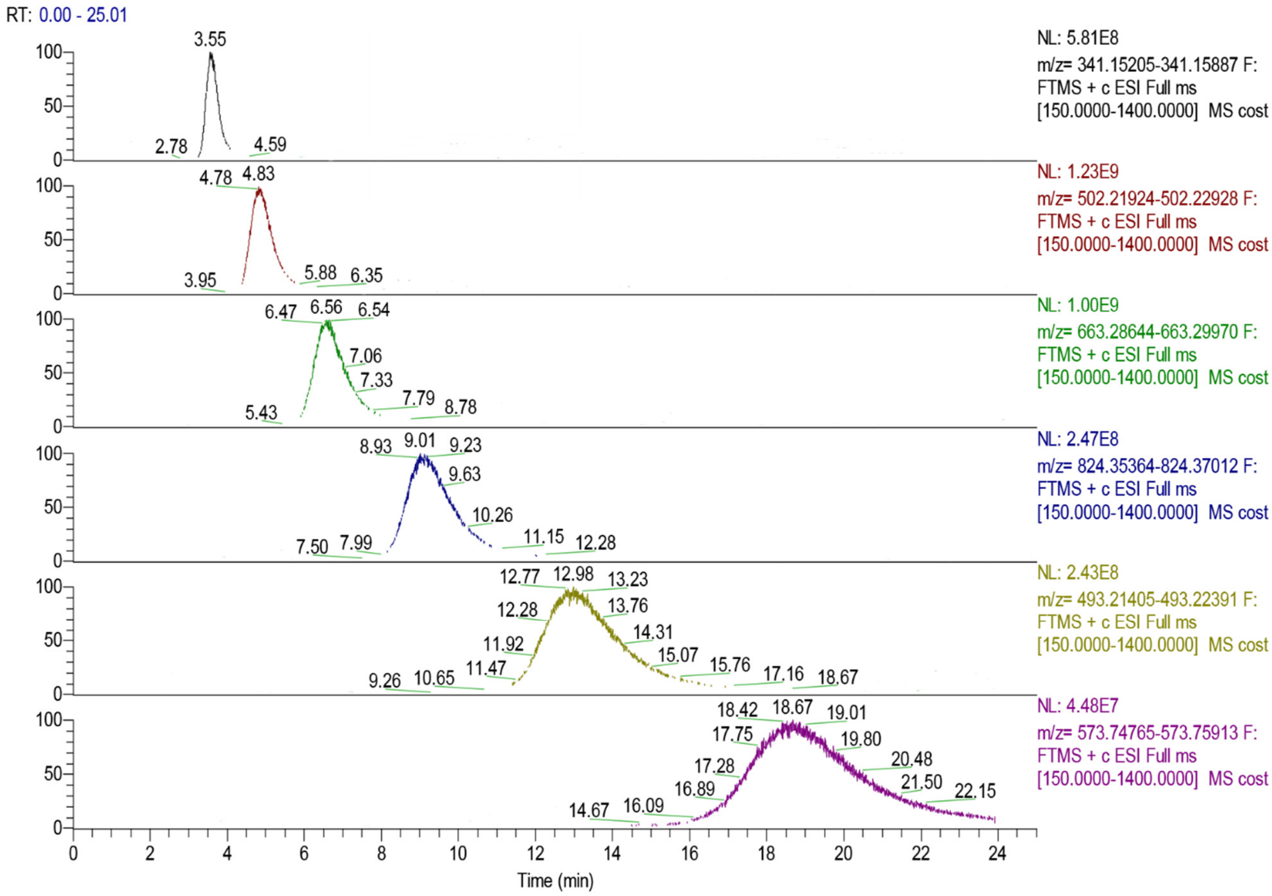

Figure S7 Liquid phase diagram of 3.440mg/mL COST

Table S1 Standard curves of retention time (RT) and concentrations of COS4, COS5 and COS6.

	**Concentration (mg/mL)**	**Apex RT**	**Start RT**	**End RT**	**Area**	**Standard curve**
COS4	0.0875	6.39	5.72	8.08	433	y=6475.3x-228.46 R^2=0.9955
	0.175	6.45	5.76	8.12	1096	
	0.35	6.39	5.68	8.08	1935	
	0.7	6.37	5.69	8.15	3946	
	1.4	6.47	5.65	8.33	9012	
COS5	0.04375	8.95	7.62	12.22	192	y=6398.6x-132.42 R^2=0.9987
	0.0875	8.81	7.65	11.91	479	
	0.175	8.90	7.65	12.39	907	
	0.35	8.81	7.69	11.87	2054	
	0.7	8.70	7.67	11.93	4384	
COS6	0.05625	12.22	10.39	17.85	222	y=5917.8x-118.04 R^2=0.9938
	0.1125	12.36	10.75	17.24	567	
	0.225	12.35	10.32	17.90	1020	
	0.45	12.67	10.62	18.36	2788	
	0.9	12.37	10.68	17.32	5132	

Table S2 Liquid phase data of COS46 and COST target components

	C/(mg/mL)	Apex RT	Start RT	End RT	Area
COS46	1.82	6.44	5.67	8.24	45242102751
		8.83	7.57	12.23	23397975217
		12.65	10.32	18.33	27828611707
COST	3.44	6.56	5.21	8.58	61663275573.503
		9.01	7.08	13.00	22671467620.854
		12.98	9.92	19.64	35211985606.695

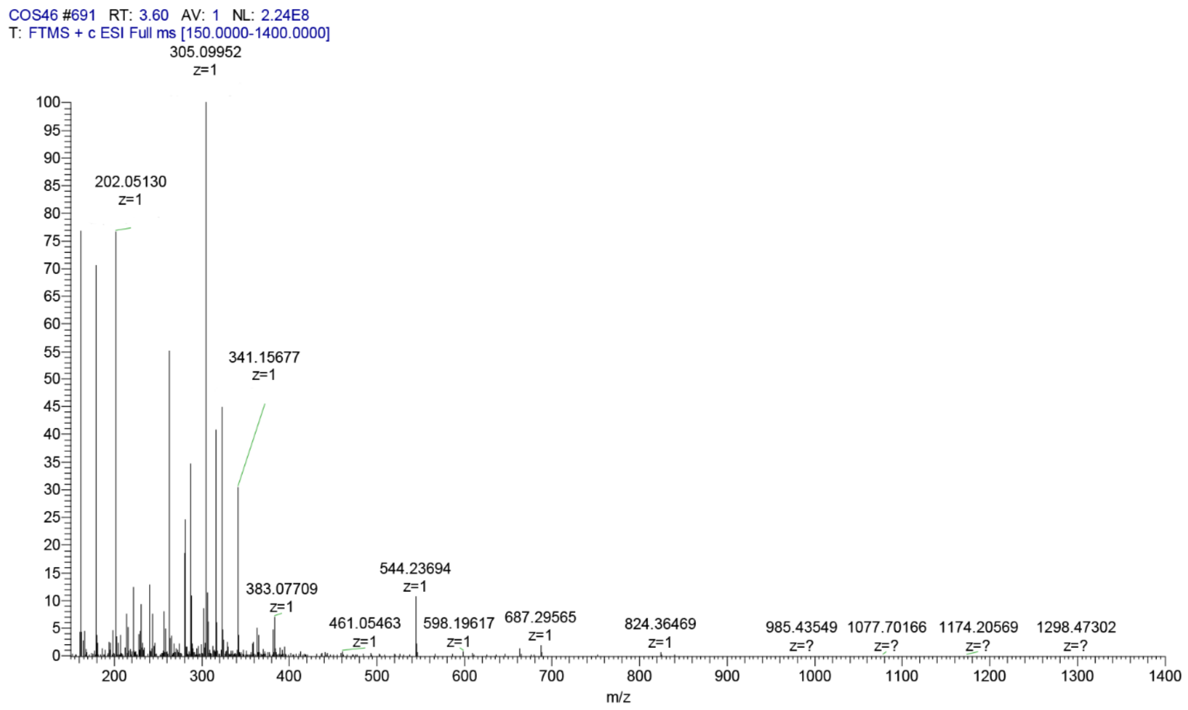

Figure S8 Mass spectrum of COS46 when RT=3.60min

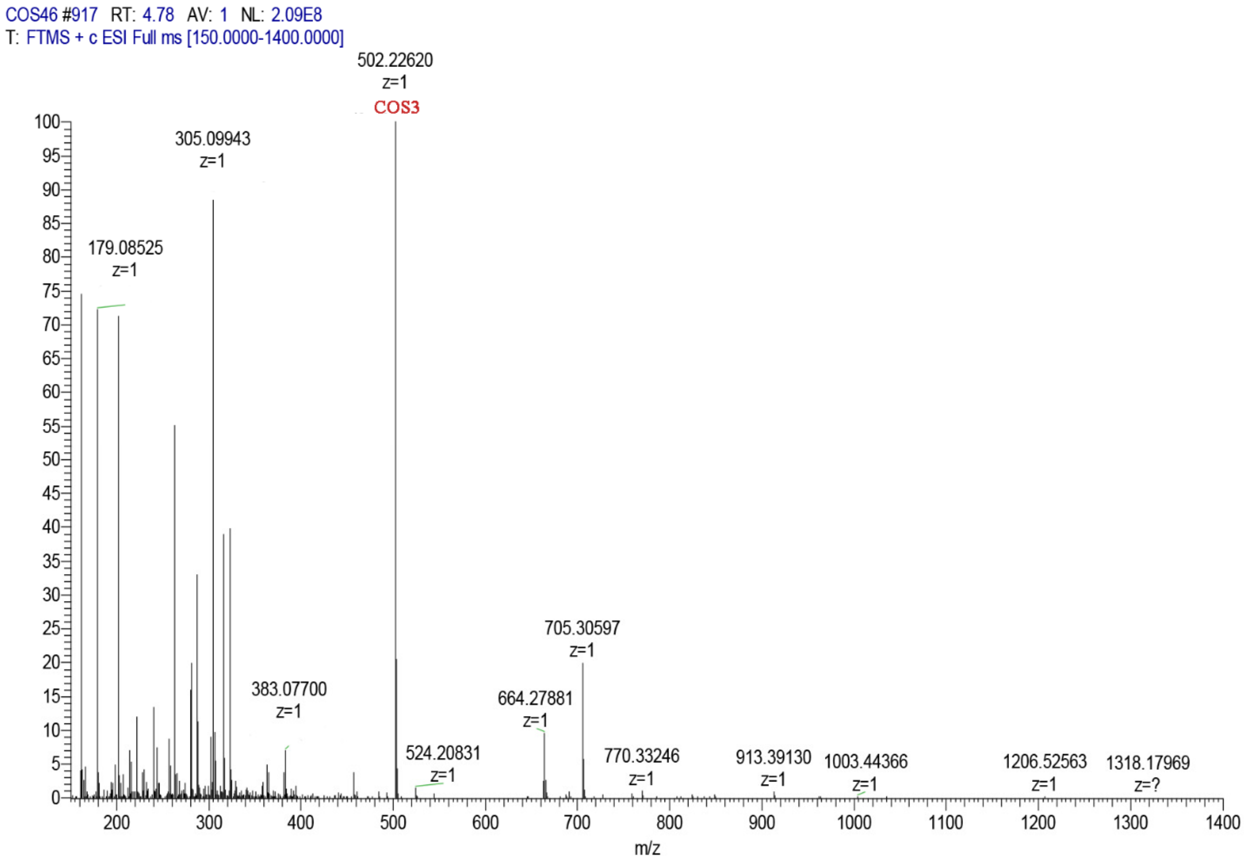

Figure S9 Mass spectrum of COS46 when RT=4.78min

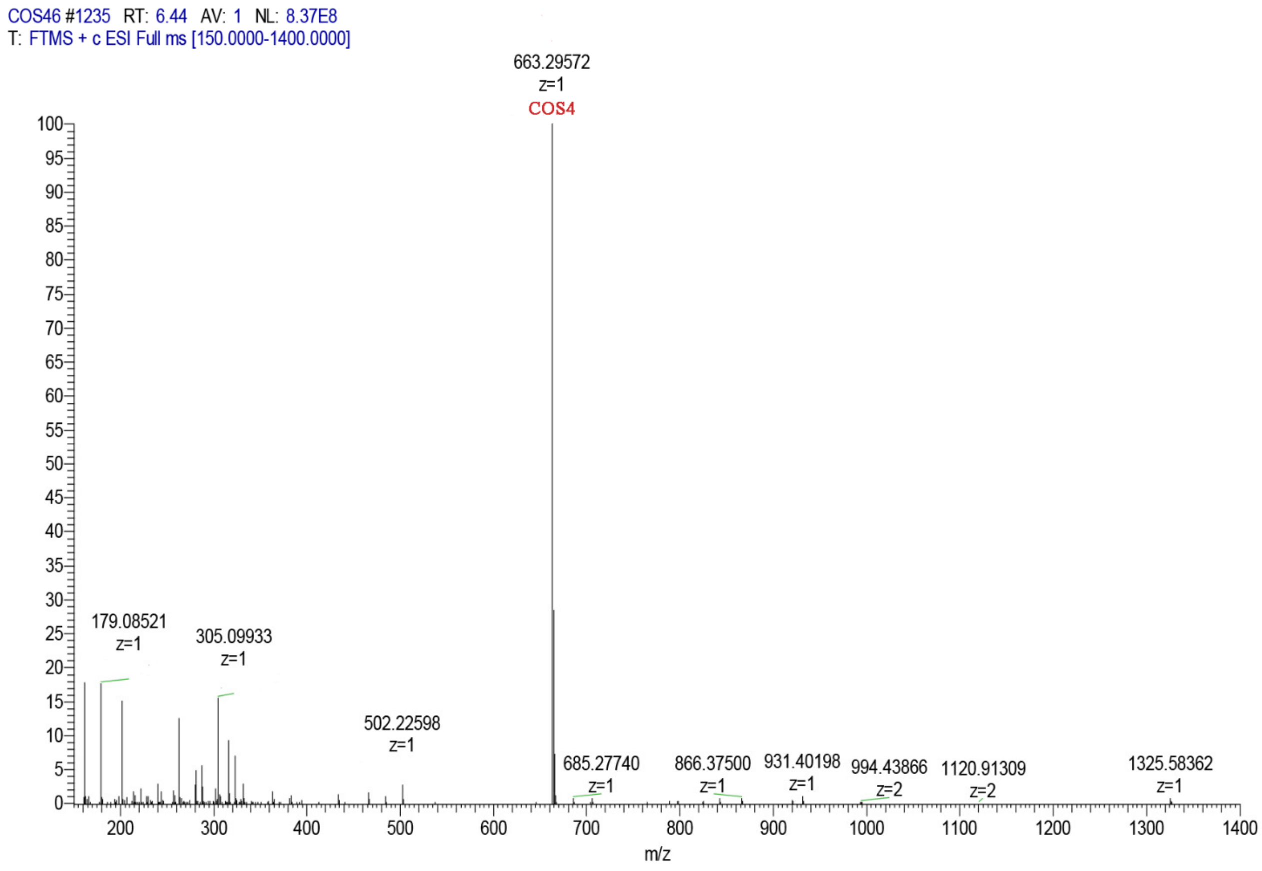

Figure S10 Mass spectrum of COS46 when RT=6.44min

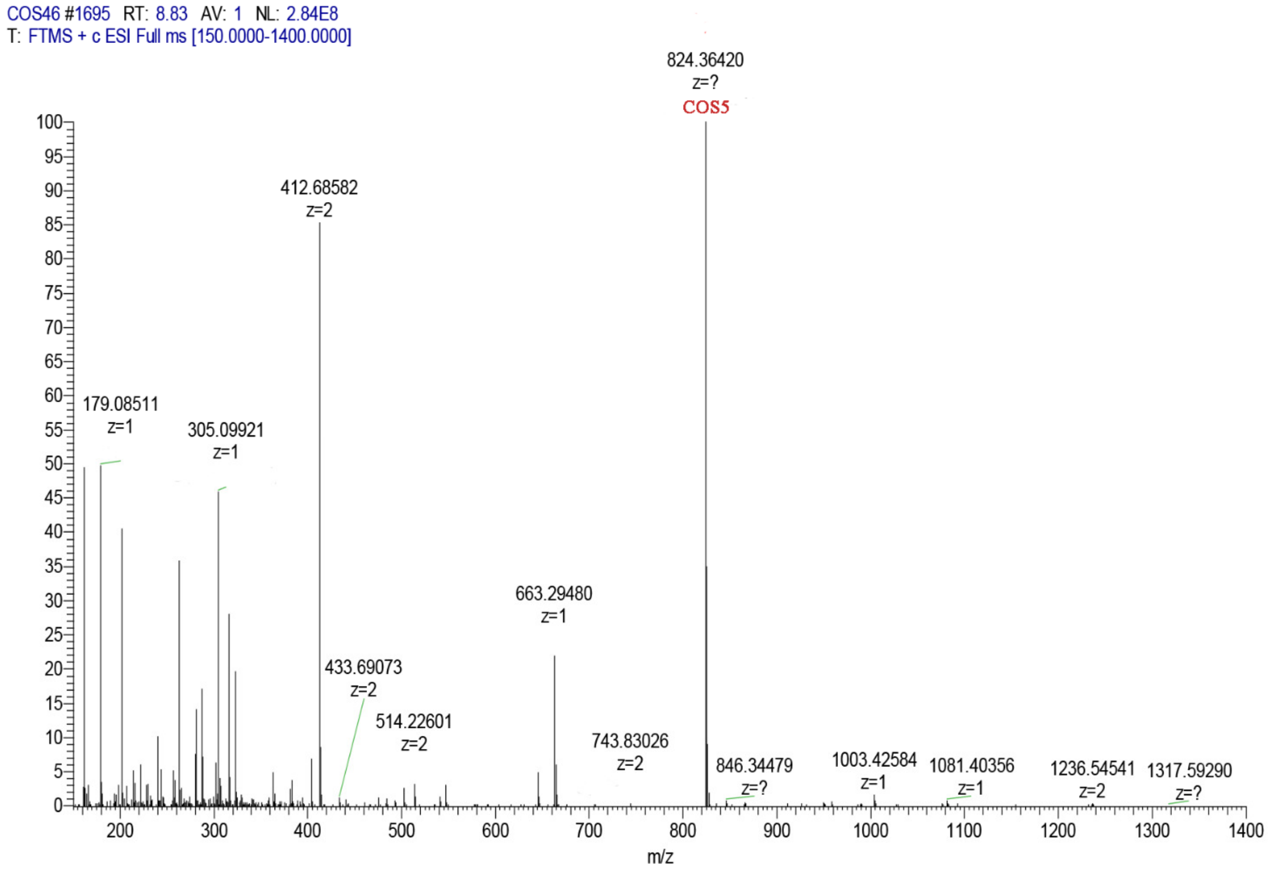

Figure S11 Mass spectrum of COS46 when RT=8.83min

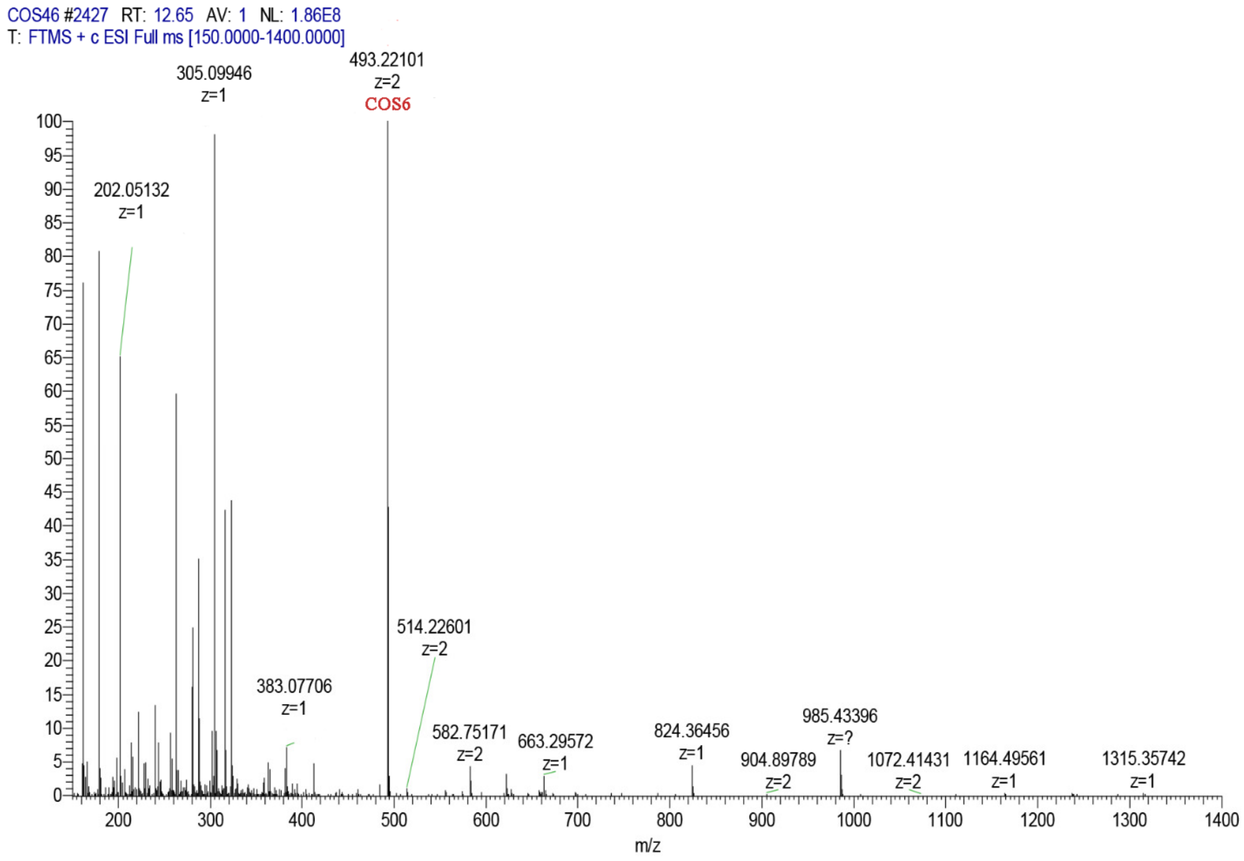

Figure S12 Mass spectrum of COS46 when RT=12.65min

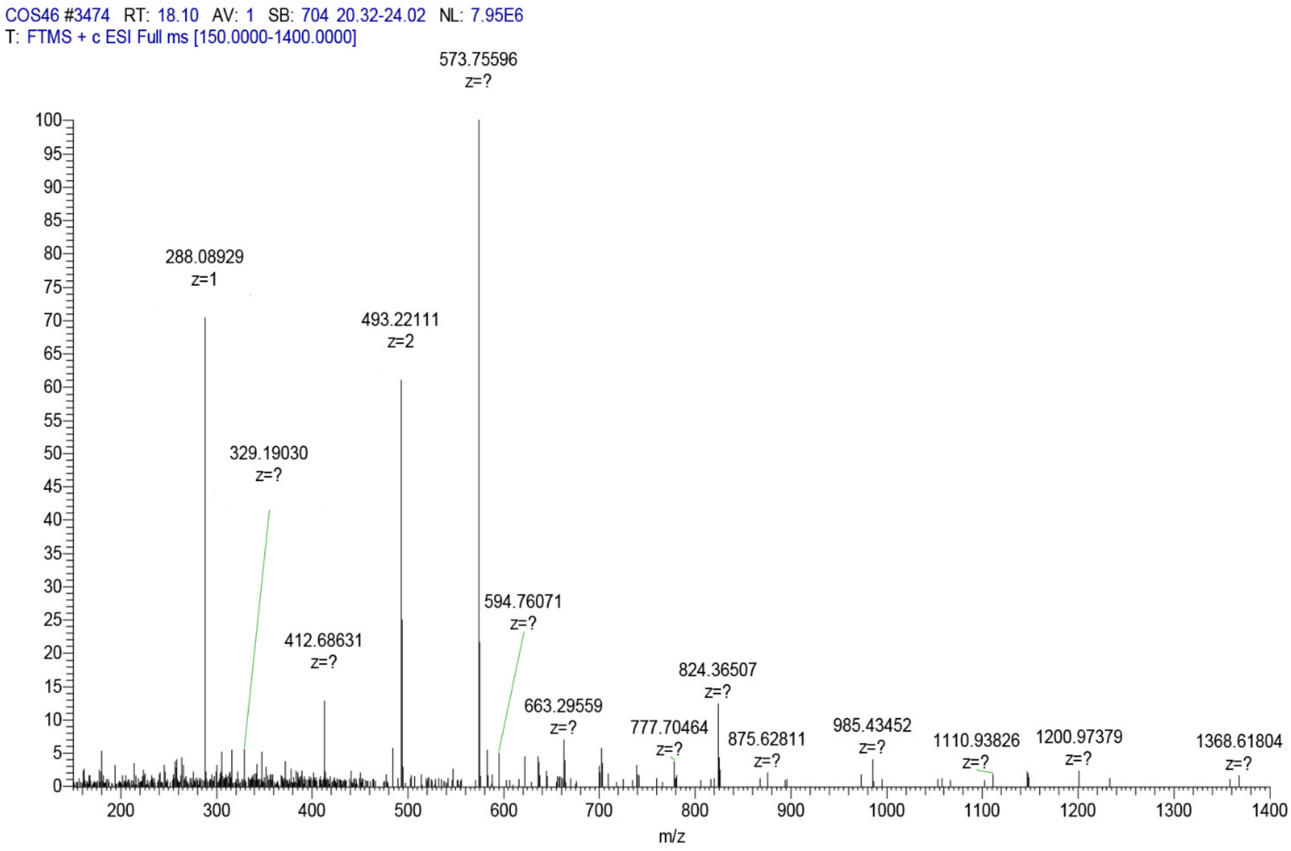

Figure S13 Mass spectrum of COS46 when RT=18.10min

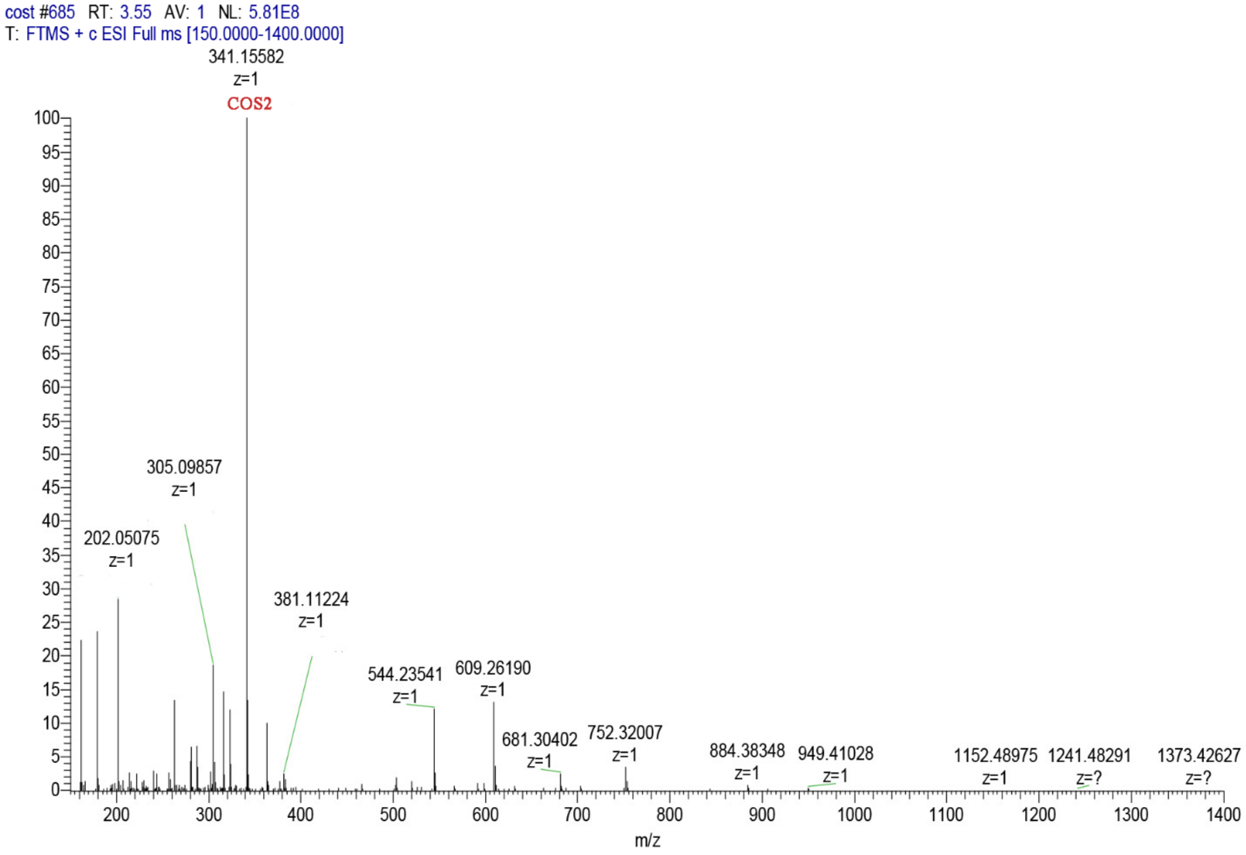

Figure S14 Mass spectrum of COST when RT=3.55min

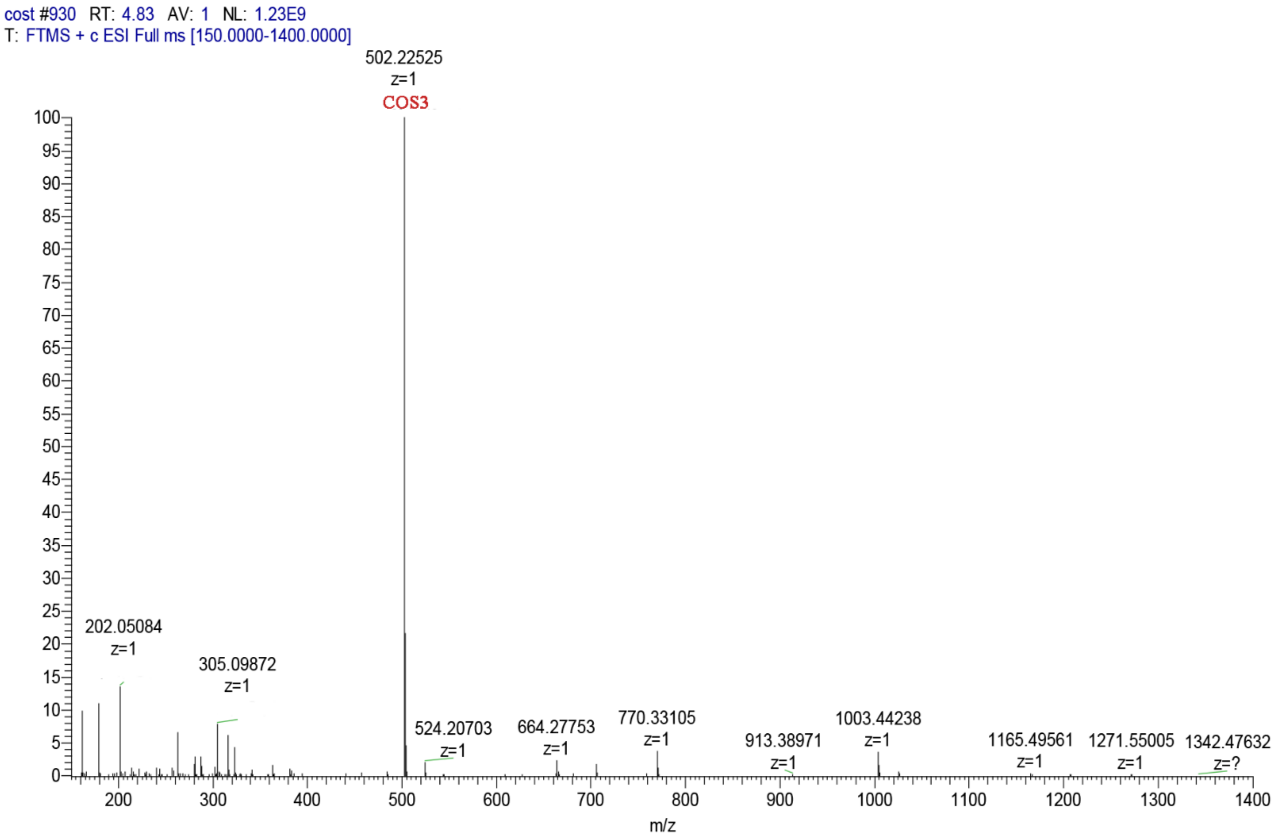

Figure S15 Mass spectrum of COST when RT=4.83min

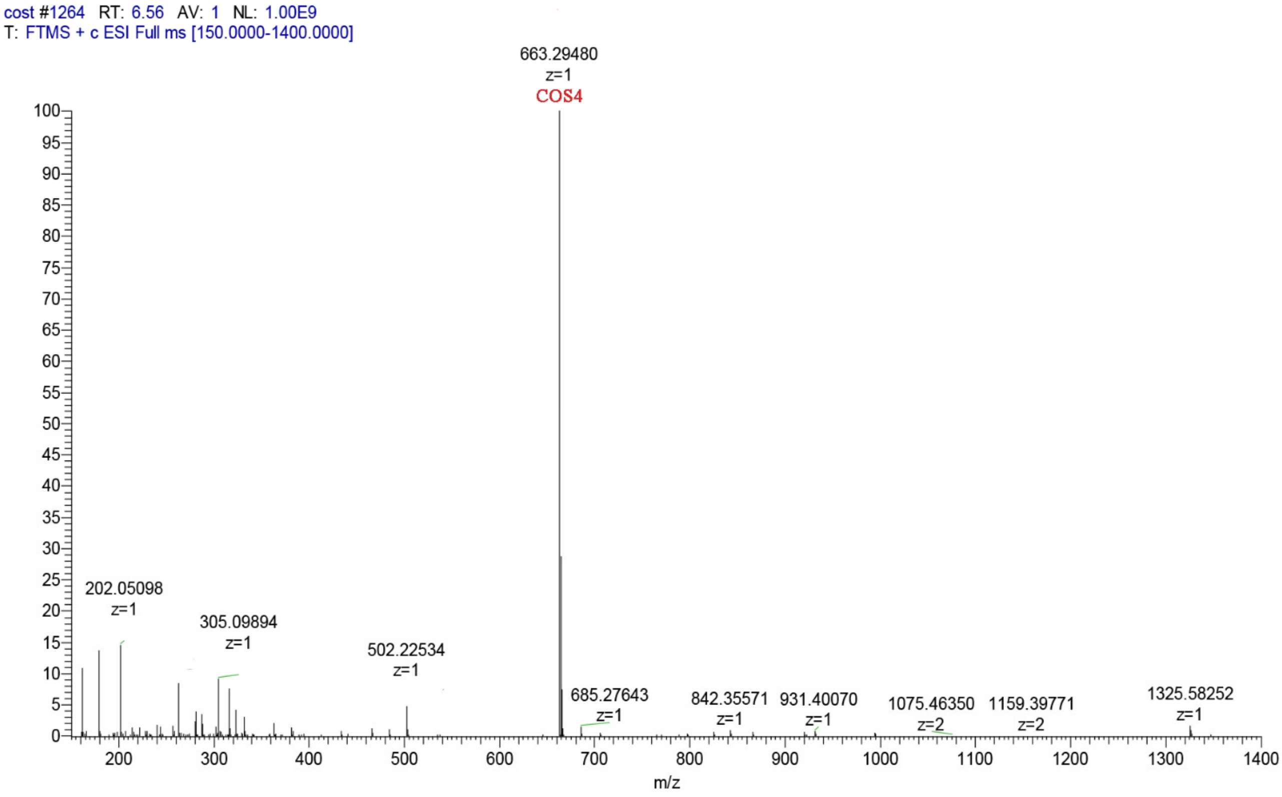

Figure S16 Mass spectrum of COST when RT=6.56min

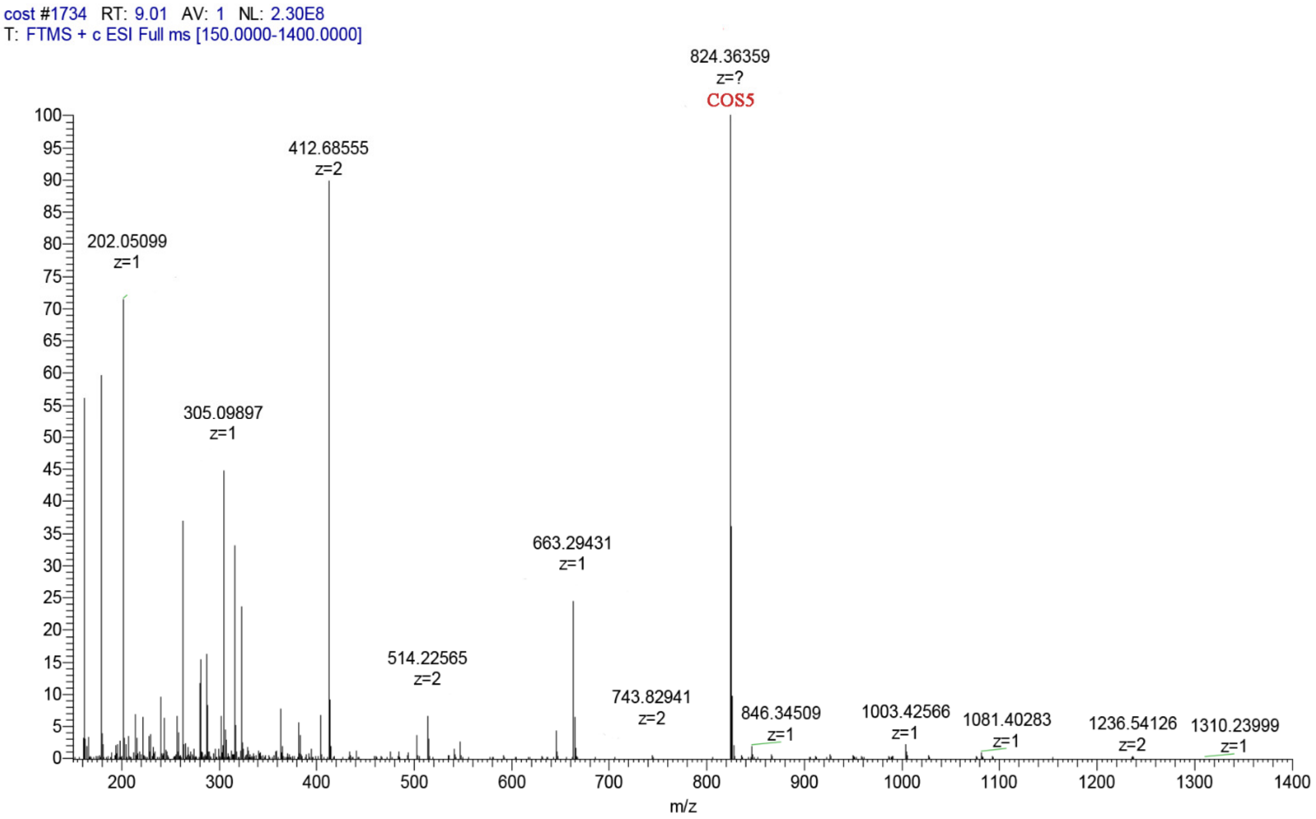

Figure S17 Mass spectrum of COST when RT=9.01min

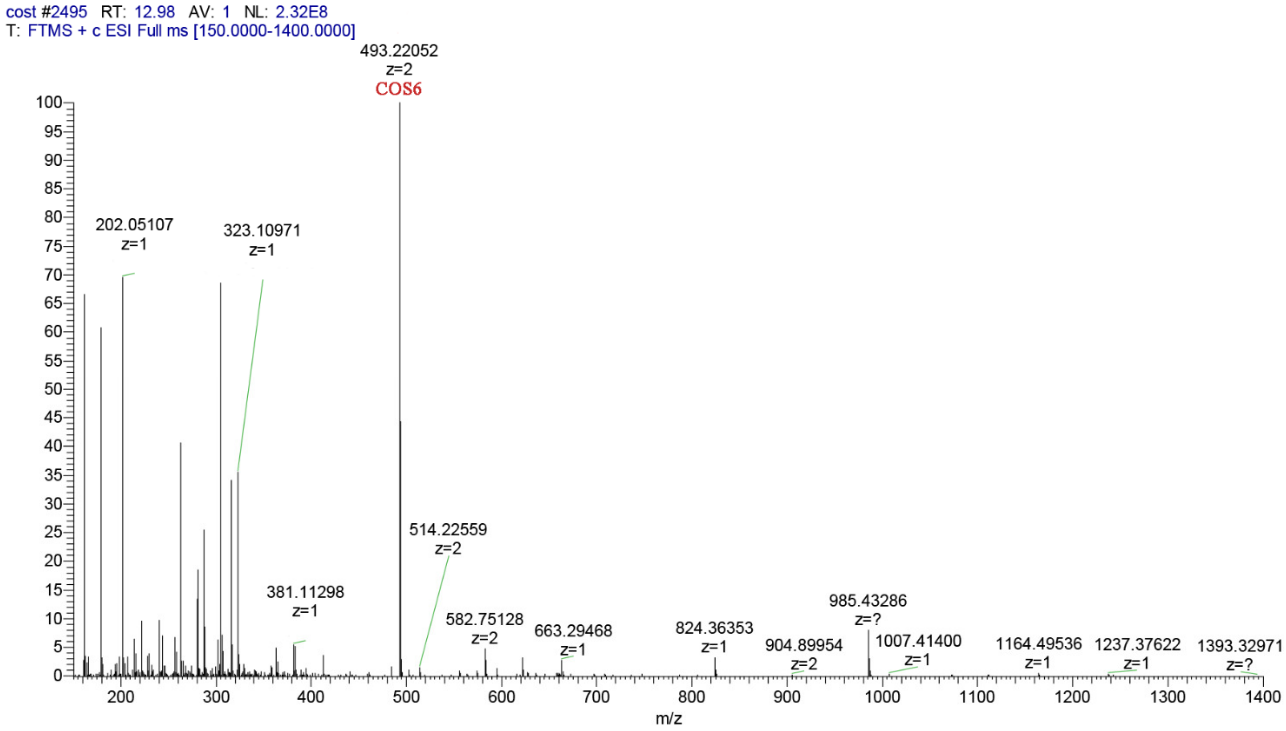

Figure S18 Mass spectrum of COST when RT=12.98min

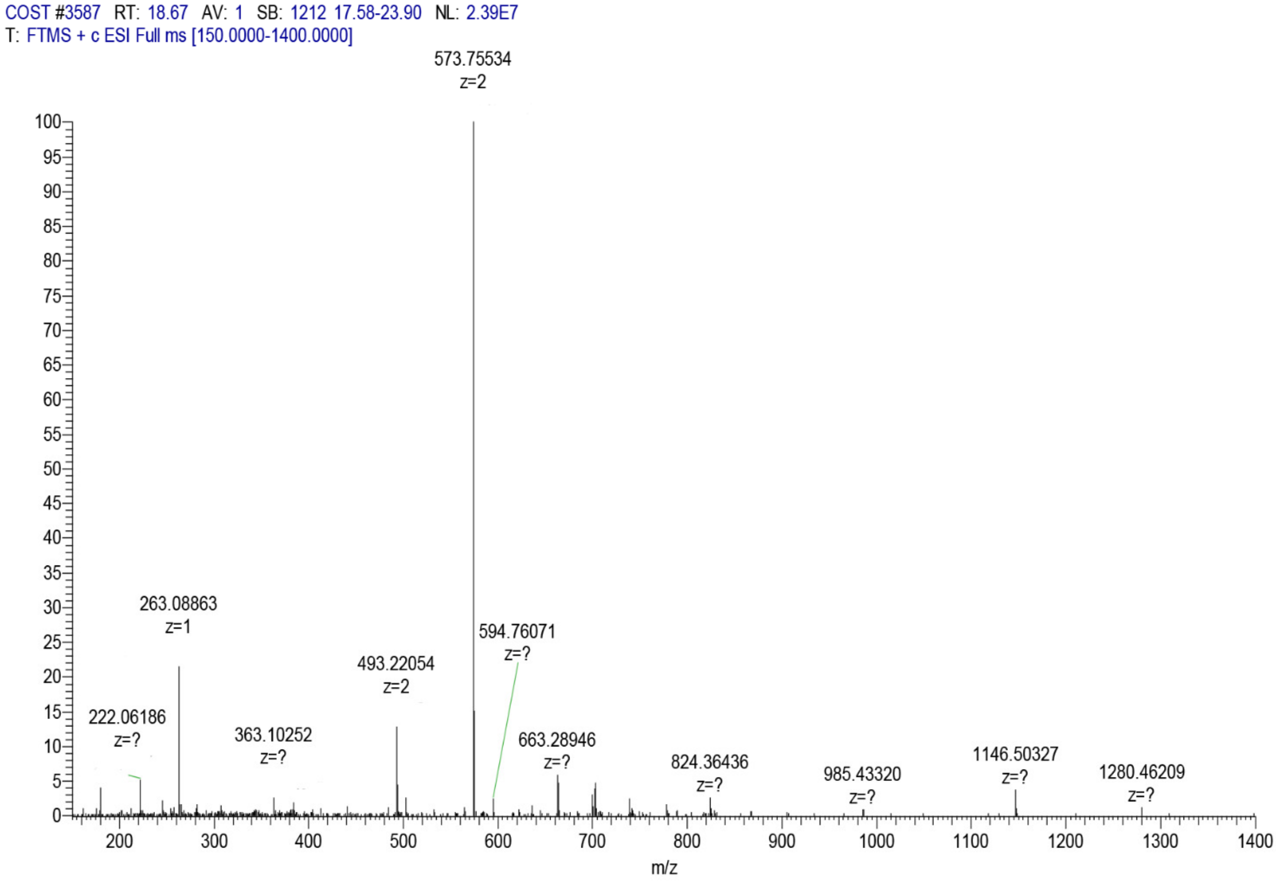

Figure S19 Mass spectrum of COST when RT=18.67min

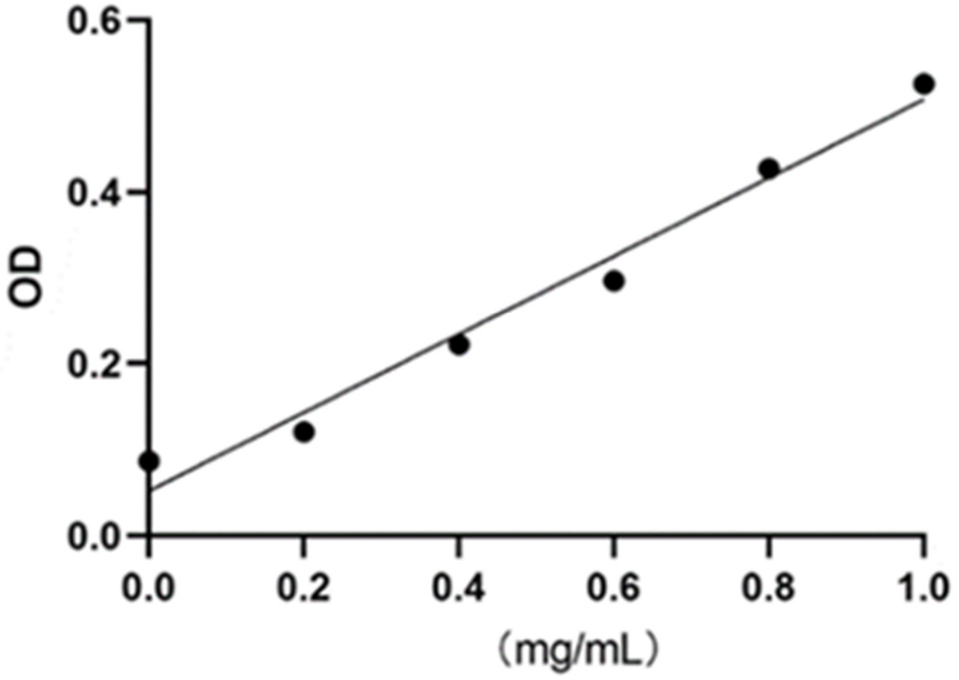

Figure S20 Standard curve of photometric method
